# Variational mode decomposition combined fuzzy—Twin support vector machine model with deep learning for solar photovoltaic power forecasting

**DOI:** 10.1371/journal.pone.0273632

**Published:** 2022-09-16

**Authors:** Gobu Balraj, Aruldoss Albert Victoire, Jaikumar S., Amalraj Victoire

**Affiliations:** 1 Department of Electrical & Electronics Engineering, Anna University, Coimbatore, Tamil Nadu, India; 2 Assistant Executive Engineer, Tamilnadu Generation and Distribution Corporation Ltd, Chennai, Tamil Nadu, India; 3 Department of Computer Applications, Sri ManakulaVinayagar Engineering College, Puducherry, India; University of Bonab, ISLAMIC REPUBLIC OF IRAN

## Abstract

A novel Variational Mode Decomposition (VMD) combined Fuzzy-Twin Support Vector Machine Model with deep learning mechanism is devised in this research study to forecast the solar Photovoltaic (PV) output power in day ahead basis. The raw data from the solar PV farms are highly fluctuating and to extract the useful stable components VMD is employed. A novel Fuzzy–Twin Support Vector Machine (FTSVM) model developed acts as the forecasting model for predicting the solar PV output power for the considered solar farms. The twin support vector machine (SVM) model formulates two separating hyperplanes for predicting the output power and in this research study a fuzzy based membership function identifies most suitable two SVM prediction hyperplanes handling the uncertainties of solar farm data. For the developed, new VMD-FTSVM prediction technique, their optimal parameters for the training process are evaluated with the classic Ant Lion Optimizer (ALO) algorithm. The solar PV output power is predicted using the novel VMD-FTSVM model and during the process multi-kernel functions are utilized to devise the two fuzzy based hyperplanes that accurately performs the prediction operation. Deep learning (DL) based training of the FTSVM model is adopted so that the deep auto-encoder and decoder module enhances the accuracy rate. The proposed combined forecasting model, VMD-ALO-DLFTSVM is validated for superiority based on a two 250MW PV solar farm in India. Results prove that the proposed model outperforms the existing model in terms of the performance metrics evaluated and the forecasted PV Power.

## Introduction

In the field of renewable energy, solar power from the sun is rapidly growing and occupying the power sector. Solar power is identified as the fastest-growing resource of electric power and world-wide the production of power from solar resource shows exponentially increase every year. The modelled solar farms across the globe substantiate the importance of solar energy and its clean source of power production. In the year 2021, based on the data from the International Renewable Energy Agency, it is inferred that the top 5 countries in solar power generation includes–China, United States, Japan, Germany and India. Table 1 details the installed megawatt capacity of solar farms and their percentage contribution of solar power across the globe. The need and importance of power generation from solar source is well lucid considering the abundance sun natural source and difficulty in handling of other forms of energy production [1–3]. Due to which, each and every country takes immense steps in building high potential solar farms and thereby to increase the rate of renewable source of power production from their country.

**Table 1:** Solar power by country– 2021 (Courtesy:MNRE).

Country	Installed Capacity MW	World Total Percentage
China	254300	35.1%
USA	75563	10.2%
Japan	66000	9.3%
Germany	53762	7.1%
India	39210	5.4%
Italy	21450	3.0%
Australia	17614	2.4%
Vietnam	16500	2.1%
South Korea	14510	2.0%
Spain	14026	2.0%

At this juncture, in respect of the supply and demand of electric power, a balance has to be achieved and therefore always there is a requirement to forecast the power production from various renewable and non-renewable energy resources. For a particular year, there exist task for each country to predict their various forms of output power so as to provide uninterrupted power supply to their people. The wide construction of solar PV farms across the globe intends to predict the solar PV output power that shall be produced from each farm and thereby the requirement of demand is to be met. Energy transition is a key factor with respect to renewables, but the rise of solar power and how cheap it has come over time is vital. In the last decade, the cost of solar energy has fallen exponentially and presently it is the cheapest mode of power generation. Also, the prediction of PV output power is vital so as to plan the various other modes of power generation and how much demand will the solar farm meets and also to synthesize the economic impact of a country.

**Fig 1:**
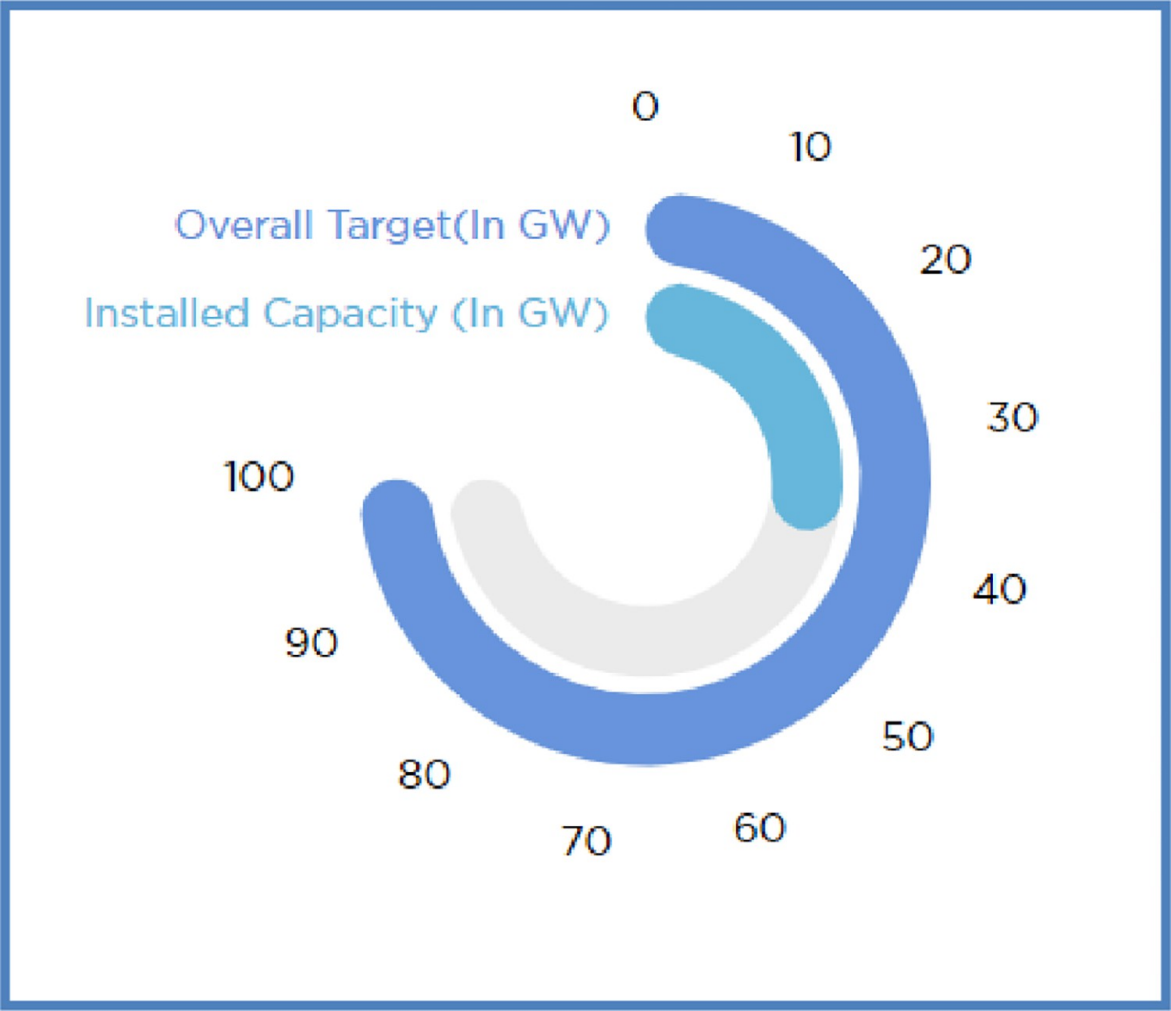
Installed solar power capacity in India– 2022 (www.mnre.gov.in).

With the vast solar energy potential in India, it is incidental to have 5000 trillion KWhour in a year and it is possible to generate power rapidly on distributed basis. Considering the aspect of energy security as well, solar energy is highly secure and available abundantly. If the solar energy is captured effectively, a very small fraction of incidental solar energy shall meet the demand of power of the entire country. On view of sustainable development, solar energy is an integral solution and play vital role in grid connected power generation. Achieving fifth global position in the world in 2021, India has raised the solar power capacity more than 10 times over the past five years and achieves better grid parity. Fig 1 shows the installed solar power capacity in GW in India for the year 2022.

From the data presented, it is well obvious that there is an increasing demand of electric power to be generated from the solar source and thus always predicting the solar PV output power based on the existing wind farms is highly essential [4]. The requirement of predicting the solar PV output power is based on the following reasons,

- Predicting solar PV output will give a notion on how much solar power shall be generated from the solar farm that spreads over a particular area
- In respect of the climatic and weather conditions, the watts of power that shall be produced from the solar energy
- It will facilitate in planning the power from other renewable and non-renewable resources
- Prediction will enhance the power engineers to plan the distribution at the grid side
- Provides an advance knowledge on the production of the solar energy so as to adjust the power production from other sources
- Predicting PV power output tends to stabilize the overall power output from the renewable energy sector
- Non-linear behaviour of the power output generation shall also be analysed

Based on the requirement of predicting solar PV output power as above, this research study focuses on developing a novel deep learning model to carry out most accurate PV output power model for the considered solar PV wind farms.

### Related works and motivations

For the past few years, numerous works have been carried out for forecasting the PV output power of solar farms across the world. Various countries Germany, United States of America, Spain, China, Japan, India, Australia and so on are involved in generating megawatts of power from the solar resource of sun. Due to meet the demand of power and maintain a balance between the supply and demand, always prediction process is carried out for the constructed solar farms so as to have a complete analysis on solar output power production and supply to the end users. Under this scenario, machine learning (ML) models are widely employed as black box models for performing the forecast mechanism of the solar PV output power [5–13] and this section of this research paper presents a detailed survey on different techniques and ML models applied over the years for predicting the PV output power.

Nespoli et al. (2022) devised a selective approach with ensemble neural models for PV power output prediction and intended to minimize the computational burden [14]. Elsaraiti and Merabet (2022) discussed a method for predicting the generated power, in the short term, of photovoltaic power plants, by means of deep learning technique based on the Long Short Term Memory (LSTM) algorithm with respect to its ability to forecast solar power data [15]. Mughal et al. (2022) developed an optimization based autoregressive neural model to do weak-ahead solar PV output prediction and evaluated the absolute percentage error [16]. Ofori-Ntow et al. (2022) modelled a novel stacked generalization methodology for prediction of long-term photovoltaic power [17]. Li et al. (2022) used back-propagation and improved Back-Propagation neural network algorithm in short-term output prediction of PV power stations [18]. Huang et al. (2022) presented a hybrid prediction model based on improved convolutional neural network and bidirectional gated recurrent unit for predicting solar generated power [19]. Akhter et al. (2022) developed a hybrid version of deep learning (DL) method (SSA-RNN-LSTM) for an hour-ahead prediction of three different PV systems [20].

Serrano Ardila et al. (2022) proposed two variants of fuzzy time series to perform short-term forecasting of solar PV generation [21]. Carneiro et al. (2022) investigated and carried out a detailed review on precise PV power and solar irradiation forecasts using physical, statistical, and machine learning models [22]. Zhang et al. (2022) proposed a gated recurrent unit neural network prediction model based on complete ensemble empirical mode decomposition for PV output power forecasting [23]. Pretto et al. (2022) modelled a novel new ensemble method based on the probabilistic distribution of the trials for photovoltaic energy production forecast [24]. Beigi et al. (2022) evaluated the ability of the neural network procedure to model and forecast solar power outputs of photovoltaic power systems with weather data [25]. Elizabeth Michael et al. (2022) developed a short-term solar irradiance prediction model called modified multi-step Convolutional Neural Network (CNN)-stacked Long-Short-Term-Memory network (LSTM) with drop-out [26]. Akhter et al. (2022) developed a deep learning approach (RNN-LSTM) to forecast the PV output power of the considered solar farms [27]. Zhang et al. (2022) performed a review of machine learning methods from different perspectives and provided a critical review of machine learning models for recent PV output power applications [28]. Yu et al. (2022) developed a convolutional long short-term memory network (CLSTM) prediction model optimized by adaptive mutation particle swarm optimization for solar power generation forecasting [29]. Ibrahim et al. (2022) introduced a new power prediction approach to enhance the power prediction quality by combining different solar models [30].

Simeunovic et al. (2021) developed two novel graph neural network models for deterministic multi-site PV forecasting dubbed the graph-convolutional long short term memory and the graph-convolutional transformer [31]. Zazoum (2022) modelled machine learning techniques such as support vector machine and Gaussian process regression to predict the power of different solar PV panel [32]. Geetha et al. (2022) employed different ANN models with three popular algorithms for predicting solar radiation and thereby the solar output power [33]. Lopes et al. (2022) employed Neural Network models for photovoltaic power forecast using remotes and local measurements [34]. Wentz et al. (2021) developed and compared the prediction accuracy of solar irradiance and PV power output between Artificial Neural Network (ANN) and Long-Term Short Memory (LSTM) network models [35]. An et al. (2021) proposed a probabilistic ensemble prediction model and tested it using two photovoltaic outputs and weather data measured from a grid-connected photovoltaic system [36]. Lee et al. (2021) explored the probabilistic approach neural model to improve the prediction of the photovoltaic rate of power output per hour [37]. Wang et al. (2021) tested the energy outputs of different types of PV modules and computed the accuracies of various simplistic PV module power prediction models [38]. Wang and Shi (2021) improved the ability of short-term solar radiation prediction using sparse subspace representation and k-nearest-neighbour approach [39]. Jiang et al. (2021) developed ultra-short-term prediction of photovoltaic (PV) output, based on an LSTM (long short-term memory)-ARMA (autoregressive moving average) combined model driven by ensemble empirical mode decomposition [40].

Abedinia et al. (2021) studied an adaptive Gaussian mixture approach and modelled a variational Bayesian model inference through multikernel regression (MkR) to assist desirable precise prediction of PV output power [41]. Zhao et al. (2021) proposed a high-precision and ultra-fast PV power prediction algorithm using Least Squares Support Vector Machine model [42]. Qu et al. (2021) proposed an attention-based long-term and short-term temporal neural network prediction model assembled using the convolutional neural network, long short-term memory neural network for day-ahead hourly photovoltaic power forecasting [43]. Mohana et al. (2021) employed machine learning (ML)-based algorithms to predict the generated power of a PV system for residential buildings [44]. Ajayi and Heymann (2021) modelled a novel Marine Predators Algorithm for both training an Artificial Neural Network model used for predicting the energy demand and PV output power [45]. Wang et al. (2021) developed two neural networks with different training ranges to replace the whole neural network for predicting I-V curves, P-V curves, and maximum power [46]. Nie et al. (2020) proposed a two-stage classification-prediction framework for predicting contemporaneous PV power output from sky images and compared it with an end-to-end convolution neural network [47]. Wang et al. (2020) presented an improved solar output power prediction method based on optimised chaotic phase space reconstruction [48]. Erduman (2020) developed an artificial neural network-based model for solar PV output power prediction [49]. Wang et al. (2020) developed an improved multi-neural network to predict the electrical characteristics of a PV module and thereby solar output power prediction under different environmental conditions [50].

Liu and Xu (2020) proposed a randomised learning-based hybrid ensemble (RLHE) model to construct the prediction intervals of probabilistic solar power output forecasting [51]. Chai et al. (2019) modelled a time learning weight to improve the time correlation of the LSTM network for PV output power prediction [52]. Gamarro et al. (2019) created a unified weather research forecasting (WRF) system called urban WRF-solar (uWRF-solar) for forecast of solar power production [53]. Douiri (2019) introduced a novel method for representing the photovoltaic (PV) characteristics using Takagi–Sugeno type neuro-fuzzy network (NF) [54]. Liu et al. (2019) investigated the effects of PV solar power variability and proposed a data-driven ensemble modelling technique for improving the prediction accuracy of PV power generation [55]. Gao et al. (2019) presented a model for PV power output forecasting using long short term memory (LSTM) networks [56].

Al-Dahidi et al. (2019) proposed an efficient Artificial Neural Network model in which 10 different learning algorithms for accurate one day-ahead PV power production predictions with short computational time [57]. Shang and Wei (2018) modelled an enhanced empirical model decomposition, a new feature selection method and an improved support vector regression for forecasting of solar power output [58]. Perveen et al. (2018) developed an intelligent fuzzy logic model based on sky-conditions for estimating global solar PV energy output so as to meet the energy requirements [59]. Lin et al. (2018) proposed a novel hybrid prediction model combining improved K-means clustering, grey relational analysis and Elman neural network (Hybrid Kmeans-GRA-Elman, HKGE) for short-term PV power prediction [60]. Preda et al. (2018) analysed data captured from loggers and forecasted the PV output with Support Vector Machine and linear regression, finding that Root Mean Square Error for prediction [61].

The growth of machine learning technique is increasing extravagantly and their applicability for solving varied problems of medical image classification, solving optimization problems and for automobile based applications has been reported in the works of Alzubi et al. (2019) [62], Braik et al. (2022) [63] and Alzubi et al. (2022) [64] respectively. Various studies in respect of IOT based solar PV based energy harvesting and based on wireless sensor networks has been dealt and is currently going on in this related field of solar PV power generation studies [65–67]. The related works section thus provides a clear insight on the works earlier and presently going on in this solar PV power production including their varied applications.

### Challenges

In view of the literature study made on the related works as above in the prediction of solar PV output power, it is lucid that several researchers has developed and analysed the machine learning based predictor models for the said application. Among the machine learning models, few feed forward models and their variants, recurrent neural predictors and memory based models has been widely used [11–18]. Also, with the growth of deep learning based techniques, researchers has initiated in developing predictor models for solar PV output power forecasting using various deep learning models for the said application [1, 14, 19, 20, 26, 27, 43, 47]. On this detailed review made on the different machine learning and deep learning models for PV output power forecasting of solar farms, they are prone to possess the disadvantages as listed below,

- Occurrences of global minima and stagnation issues [3–7]
- Scalability problems on the normalization procedures adopted [2, 8, 12–17]
- Over-fitting and under-fitting issues [5, 6, 9–11, 23, 48, 51]
- Dimensionality constraints of the solar farm data and data handling issues [18–24]
- Elapsed training time [29, 31, 37]
- Data extraction problems in regression based ML models [10–15]
- Higher number of trainable parameters in DL models [1, 14, 19–20, 26, 27, 43, 47]
- Repetitive training of deep neural networks [19, 20, 26, 27]
- High computational overhead due to repetitive process [29–36]
- Few predictor models with high complexity and data redundancy [45–49]
- Difficulty in handling various forms of data [53, 58–60]
- Curse of dimensionality issues [39–42]
- Some of the techniques had difficulty in handling the variations in data scale [44]
- Reliability and stability of neural models [59]

### Need for the proposed approach

Under these circumstances, the motivation of this research study is to develop, design and simulate a novel hybrid deep learning neural predictor model for forecasting the solar PV output power for the considered solar PV farms. Based on the need and the demand of power, this work is highly motivated based on generating more power from the solar energy resources [68, 69] and thereby this prediction process will facilitate in planning the overall requirement of energy from various sources and hence the end users shall be benefitted. Considering all these limitations of the existing works and the need for solar PV power generation, the need for proposed approach for prediction of PV power includes,

- To predict how much power will be produced from the specified range of PV farms in an accurate manner
- The predicted power value will help the power engineers to plan for the output to be delivered from a particular plant, so that grid capacity shall be planned.
- To overcome the existing overheads and complexities in the present prediction models
- Will help the power engineers working in renewable energy sector in facilitating the required power generation from various forms

To handle all the limitations of local and global optima, under-fitting and over-fitting issues, premature and delayed convergence of existing predictor models, this suggested is proposed and to operate in most accurate prediction for enhancing the planning of the required power generation sector [70–73]

### Contributions of research study

Forecasting of solar PV output power from the solar farms is of prime importance so as to stabilize and have advance knowledge on the overall power output from the renewable energy sector. The prediction will also help the power engineers to analyse the non-linear behaviour of the generated output power. In this aspect, the main contributions of the research study includes,

- Employing the variational mode decomposition (VMD) for decomposing the data and to overcome the higher fluctuations in the data and as well to extract the useful components.
- Developing the hybrid form of fuzzy–twin support vector machine (FTSVM) to perform the prediction process by formulating the two hyper decision planes and enhance the prediction accuracy.
- Devising a suitable fuzzy membership function to handle data uncertainty and also in the applicability of multi-kernel functions to attain perfect prediction
- Applicability of deep learning based architecture design of the FTSVM and developing a DLFTSVM predictor thereby achieving higher accuracy rate during the prediction process.
- Adopting Ant Lion Optimizer (ALO) to attain optimal learning parameters for the proposed DLFTSVM model.
- Testing and validating the developed VMD-ALO-DLFTSVM model for two 250 MW solar farms in India.

## Methods and materials

This sections of the paper details the development of the proposed DLFTSVM predictor model and also describes the basic operation of data decomposition using VMD and the basic ALO algorithm. The PV datasets pertaining to the solar farms at the considered location is also detailed in this section.

### Data decomposition–VMD technique

A state of the art decomposition technique proposed by Dragomiretskiy and Zosso (2013) [74] is the variational mode decomposition and here the considered solar PV farm data is a time series data *p(t)* and it gets decomposed into discrete number of modes *m*_*q*_*(t)*. The decomposition is done by maintaining the sparsity features and Hilbert transform is applied to identify the central frequency*γ*_*q*_ corresponding to the bandwidth *BW(m*_*q*_*(t))*. The decomposition is executed in such a way that during reconstruction of all the decomposed modes results in the original time series data. Considering the time series data *p(t)*, it gets decomposed into numerous set of modes *m*_*q*_*(t)*, *q = 1*,*2*,*3*,*…*,*Q*, with *Q* as the total number of modes.


$$m_{q}(t)=S_{q}(t)\cos(\omega_{q}(t))$$ (1)

In Eq (1), *S*_*q*_*(t)* indicates the non-negative region of envelope and *ω*_*q*_*(t)* specifies the non-decreasing phasor function. The procedure adopted to decompose signal employing VMD is given by,

Step 1: Hilbert transform determines the signal *m*_*q*,*S*_*(t)* for each *m*_*q*_*(t)*mode and its unilateral spectrum is formed with,

$$\begin{array}{l} Hm_{q}(t)=\frac{1}{\pi }p.v\underset{R}{\int }\frac{m_{q}(y)}{t-y}dy\\ m_{q,S}(t)=m_{q}(t)+jHm_{q}(t)=S_{q}e^{j\omega_{q}(t)} \end{array}$$ (2)

Step 2: In respect of each mode *m*_*q*_*(t)*, the frequency spectrum gets shifted based on its base band and is given by,

$$m_{q,S}(t)=m_{q,S}(t)e^{-j\omega_{q}(t)}$$ (3)

Step 3: For signal in Eq (3), the bandwidth of the signal is attained with the gradient of the L^2^-norm,

$$BW(m(q))={\left\|\partial_{t}[\left(\delta (t)+\frac{j}{\pi t})*m_{q}(t\right)]e^{-j\gamma_{q}t}\right\|}_{2}^{2}$$ (4)

Step 4: The variational decomposition problem is defined to be,

$$\underset{m_{q},\gamma_{q}}{\min}\{\sum_{q=1}^{Q}{\left\|\partial_{t}[\left(\delta (t)+\frac{j}{\pi t})*m_{q}(t\right)]e^{-j\gamma_{q}t}\right\|}_{2}^{2}\}\;subject\;to\sum_{q=1}^{Q}m_{q}=p(t)$$ (5)

Where, *δ*(*t*) represents the Dirac distribution.

Step 5: For the variational problem presented in Eq (5), its solution is evaluated using the Lagrangian multiplier as given by,

$$\begin{array}{l} LM(\{m_{q}\},\{\gamma_{q}\},\beta )≔\alpha \sum_{q}{\left\|\partial_{t}[\left(\delta (t)+\frac{j}{\pi t})*m_{q}(t\right)]e^{-j\gamma_{q}t}\right\|}_{2}^{2}+\\ \;{\left\|p(t)-\sum_{q}m_{q}(t)\right\|}_{2}^{2}+\langle \beta (t),p(t)-\sum_{q}m_{q}(t)\rangle \end{array}$$ (6)

In Eq (6), reconstruction accuracy is retained with a penalty factor *α* and *β(t)* models the variational problem as the dual unconstrained problem. Eq (5) shall be solved by finding the saddle point of Eq (6).

This VMD procedure is adopted in this research study to decompose the solar PV time series data and obtain the discrete frequency components and carry out the deep learning based prediction with these components as inputs.

### Ant lion optimizer–Revisited

In view of the hunting behaviour of the ant lions, a nature inspired algorithm modelled was the ant lion optimizer (ALO) by Mirjalli (2015) [75, 76]. The foraging behaviour of hunting in larvae phase and reproductive behaviour in adult phase forms the ALO approach. Their capability to dig a pit with their jaws and making the ants to get trapped into it, is employed to model the trapping of solutions. The ant lion digs the trap of particular size based on its hunger level and size of moon. The ALO algorithm is devised based on the random movements of ants, constructing traps; ants falling in traps, catching the prey have and further reconstructing the traps. The ant’s position (*P*_*ants*_) and fitness (*F*_*ants*_) are given to be,

$$P_{(n,d)ants}=(\begin{array}{cccc} P_{1,1}. & P_{1,2.} & .... & P_{1,n}\\ P_{2,1} & P_{2,2} & .... & P_{2,d}\\ . & . & . & .\\ P_{n,1} & P_{n,2} & .... & P_{n,d} \end{array})\;F_{(n,d)ants}=(\begin{array}{l} \;f(\left|P_{1,1},P_{1,2},\ldots \ldots ,P_{1,d}\right|)\\ \;f(\left|P_{2,1},P_{2,2},\ldots \ldots ,P_{21,d}\right|)\\ .\\ .\\ .\\ \;f(\left|P_{n,1},P_{n,2},\ldots \ldots ,P_{n,d}\right|) \end{array})$$ (7)

In Eq (7) ‘*f(P)*’ represents the fitness function for evaluation, ‘*n*’ is the population of number of ants, ‘*d*’ indicates the dimension. The ant lion’s position (*P*_*ant_lion*_) and fitness (*F*_*ant_lion*_) are given by,

$$P_{(n,d)ant\_lion}=(\begin{array}{cccc} L_{1,1}. & L_{1,2.} & .... & L_{1,n}\\ L_{2,1} & L_{2,2} & .... & L_{2,d}\\ . & . & . & .\\ L_{n,1} & L_{n,2} & .... & L_{n,d} \end{array})\;F_{(n,d)ant\_lion}=(\begin{array}{l} \;f(\left|L_{1,1},L_{1,2},\ldots \ldots ,L_{1,d}\right|)\\ \;f(\left|L_{2,1},L_{2,2},\ldots \ldots ,L_{21,d}\right|)\\ .\\ .\\ .\\ \;f(\left|L_{n,1},L_{n,2},\ldots \ldots ,L_{n,d}\right|) \end{array})$$ (8)

The random walk of ants with step size ‘*t*’ is given by,

$$Y(t)=[0,\sum 2y(t_{1})-1,\sum 2y(t_{2})-1,....,\sum 2y(t_{n})-1,\ldots \ldots ,\sum 2y(t_{n})-1]$$ (9)

The position update equation pertaining to the ants is,

$$P_{n\_pos}^{t}=\frac{\left(P_{n\_pos}^{t}-x_{np})(z_{np}-q_{np}^{t}\right)}{\left(r_{np}^{t}-x_{np}\right)}+q_{t}$$ (10)

In Eq (10), ‘*x*_*np*_’ and ‘*z*_*np*_’ indicates the minimum and maximum walk of ants and $q_{np}^{t}$, $r_{np}^{t}$ represent the minimum and maximum n-th variable. The trap of ants (solution) is provided using,

$$\begin{array}{l} q_{np}^{t}=L_{np}^{t}+q^{t}\\ r_{mp}^{t}=L_{np}^{t}+r^{t} \end{array}$$ (11)

The ants sliding into the pits dig and thereby moving toward optimality,

$$q^{t}=\frac{q^{t}}{\sigma },r^{t}=\frac{r^{t}}{\sigma }$$ (12)

and $\sigma =10^{\phi (\frac{t}{Q})}$, ‘*t*’ is the present iteration, ‘Q’ specifies the maximum number of iteration and ‘*ϕ*‘ gives constant values between 2 to 6. The ant lion catches the prey ant on reaching the bottom of the pit and then it consumes. The ant updates its position for catching its new prey and its equation becomes.


$$L_{np}^{t}=P_{np}^{t}\;if\;f(P_{np}^{t})>f(L_{np}^{t})$$ (13)

### Proposed VMD-ALO based deep fuzzy–Twin support vector machine model

A combined model with the decomposition technique, optimizer and deep learning approach is developed in this research study to predict the solar PV output power for the considered solar farm sites. The combined approach is VMD-ALO-DLFTSVM model and here fuzzy based twin decision hyperplanes are formulated to identify the respective classes and thereby better prediction accuracy is achieved.

### Twin SVM model

A model that formulates a two non-parallel hyperplanes by finding solutions to two quadratic optimization problems is the twin support vector machine model and these two hyperplanes are capable of categorizing the one close to the respective classes and the other that is far away from one another [77, 78]. The two non-parallel hyperplanes formulated with TSVM is,

$$\begin{array}{l} w_{+}^{T}y+w_{0+}=0\\ w_{-}^{T}y+w_{0-}=0 \end{array}$$ (14)

With respect to Eq (14) of deriving the two hyperplanes, the quadratic optimization problem is defined as,

$$\begin{array}{l} \underset{w_{+},w_{0+},\delta_{-}}{\min}\frac{1}{2}\|Pw_{+}+v_{+}w_{0+}\|+k_{1}v_{-}^{T}\delta_{-}\;such\;that-(Qw_{+}+v_{-}w_{0+})\geq v_{-}-\delta_{-},\delta_{-}\geq 0\\ \underset{w_{-},w_{0-},\delta_{+}}{\min}\frac{1}{2}\|Qw_{-}+v_{-}w_{0-}\|+k_{2}v_{+}^{T}\delta_{+}\;such\;that(Pw_{-}+v_{+}w_{0-})\geq v_{+}-\delta_{+},\delta_{+}\geq 0 \end{array}$$ (15)

In Eq (15), ‘*k*_*1*_’ and ‘*k*_*2*_’ represents the tuning parameters, the dimensional vectors are *v*_*+*_and *v*_*-*_ and *P* and *Q* specifies the matrices of the labelled classes pertaining to the elements. The algorithm intends to determine two hyperplanes, one corresponding to the near prediction category and the other far away from the prediction category. Due to which, the predicting samples coordinates to which hyperplane it shall get categorized and is closer to. For Eq (15), the fitness function attains class +1 w.r.to the hyperplane $w_{+}^{T}y+w_{0+}=0$, and to the class -1 w.r.to the hyperplane $w_{-}^{T}y+w_{0-}=0$. Now applying Lagrange multipliers to obtain the dual optimization problem as,

$$\begin{array}{l} \max\;v_{-}^{T}\alpha -\frac{1}{2}\alpha^{T}R{\left(S^{T}S\right)}^{-1}R^{T}\alpha \;such\;that\;0\leq \alpha \leq k_{1}\\ \max\;v_{+}^{T}\beta -\frac{1}{2}\beta^{T}G{\left(H^{T}H\right)}^{-1}G^{T}\beta \;such\;that\;0\leq \beta \leq k_{2} \end{array}$$ (16)

The solution from Eq (16) attains the two proximal hyperplanes,

$$\begin{array}{l} u_{+}^{T}=(w_{+}^{T},w_{0+})=-{\left(S^{T}S+\mu I\right)}^{-1}R^{T}\alpha \\ u_{-}^{T}=(w_{-}^{T},w_{0-})=-{\left(H^{T}H+\mu I\right)}^{-1}G^{T}\beta \end{array}$$ (17)

Regularization term *μI* is introduced in Eq (17) to handle the singularity and non-linear occurrences of *S*^*T*^*S* and *H*^*T*^*H*, and *I* specifies the identity matrix of suitable dimensions.

**Fig 2:**
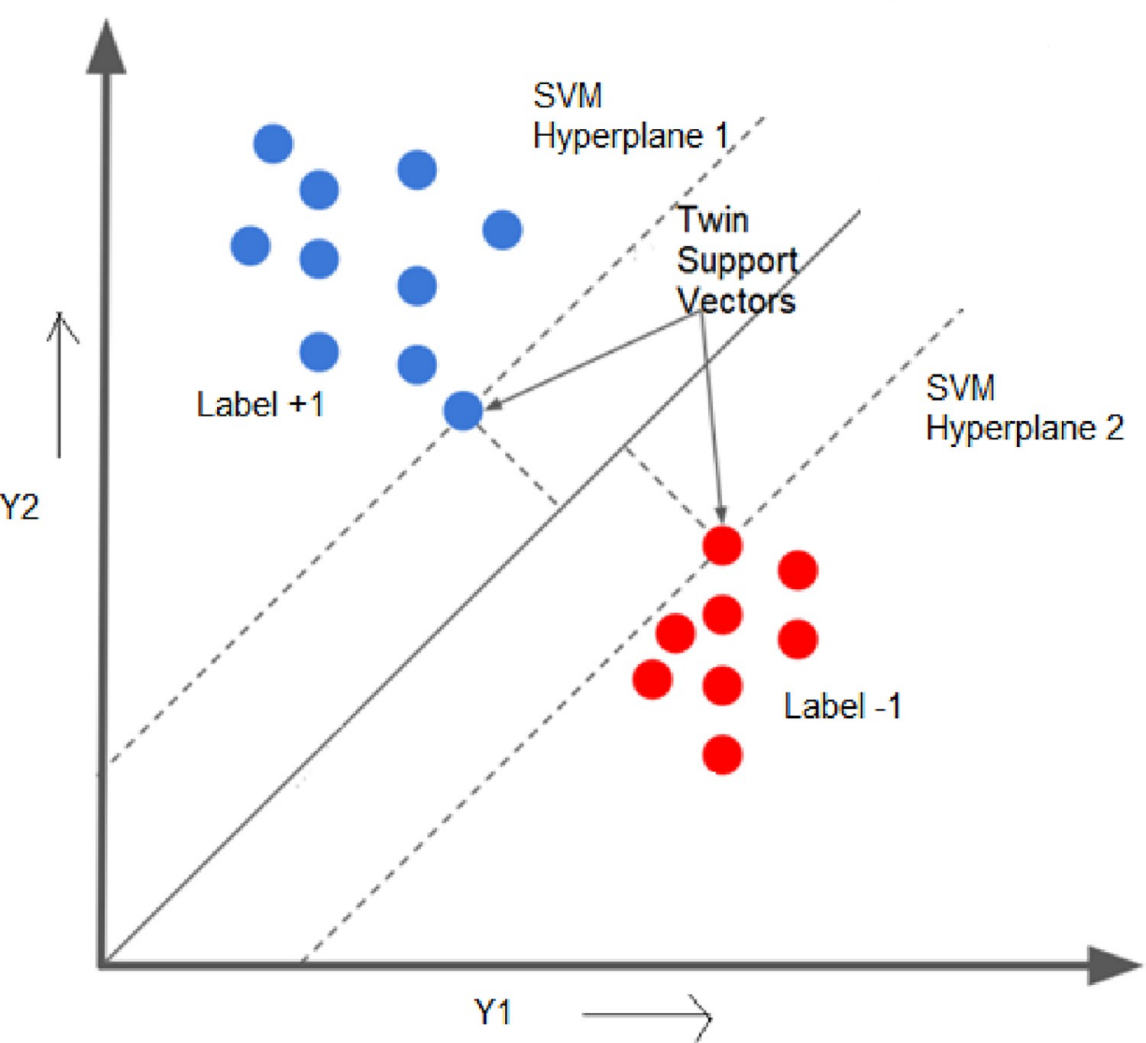
Twin support vector machine predictor model.

Fig 2 provides the presence of twin hyperplanes in the defined hyperspace depicting the operation of the TSVM technique. The algorithm tends to devise most appropriate two hyperplanes and thereby performs the prediction process.

### Proposed VMD-ALO-DLFTSVM predictor

**Fig 3:**
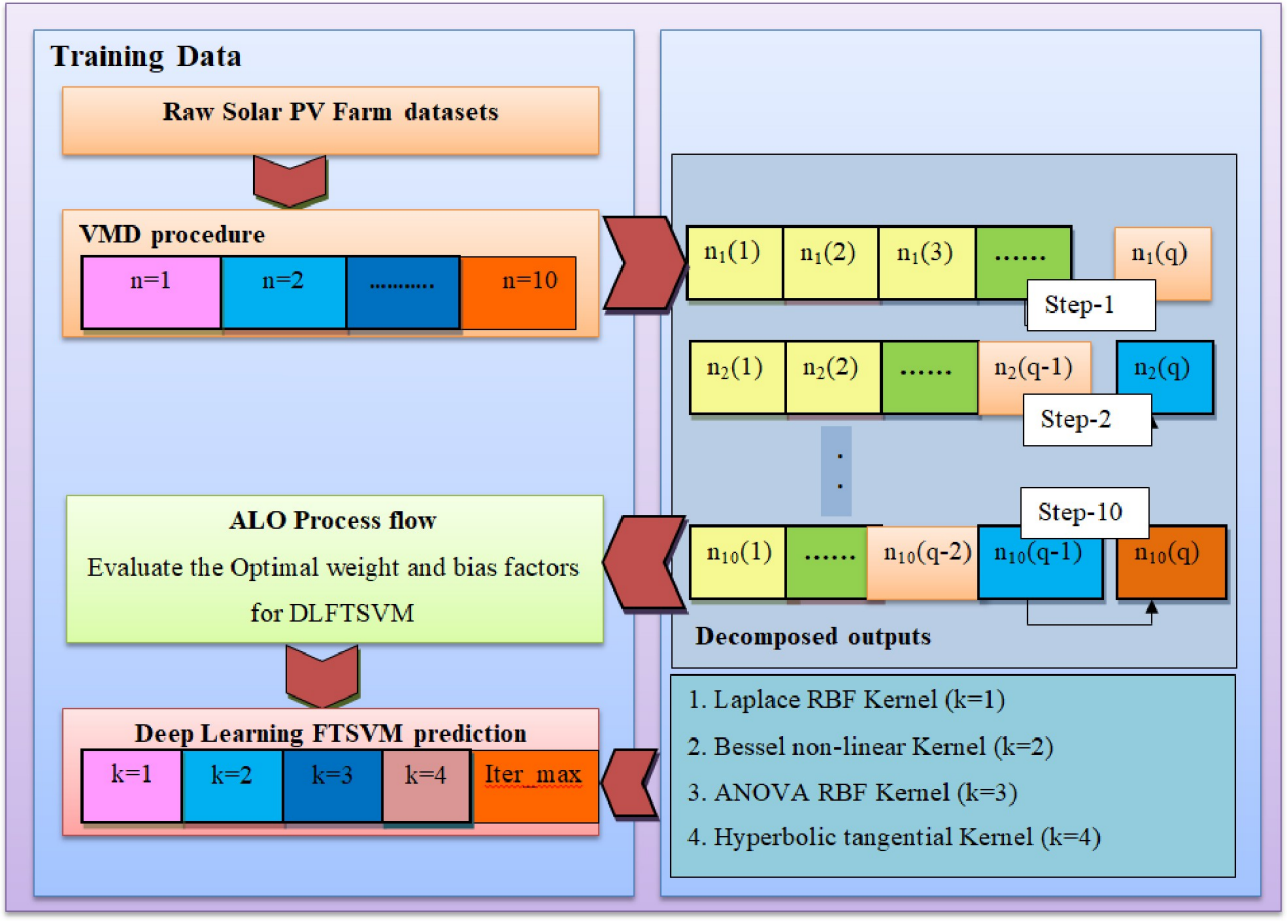
Proposed VMD-ALO-DLFTSVM predictor framework.

**Fig 4:**
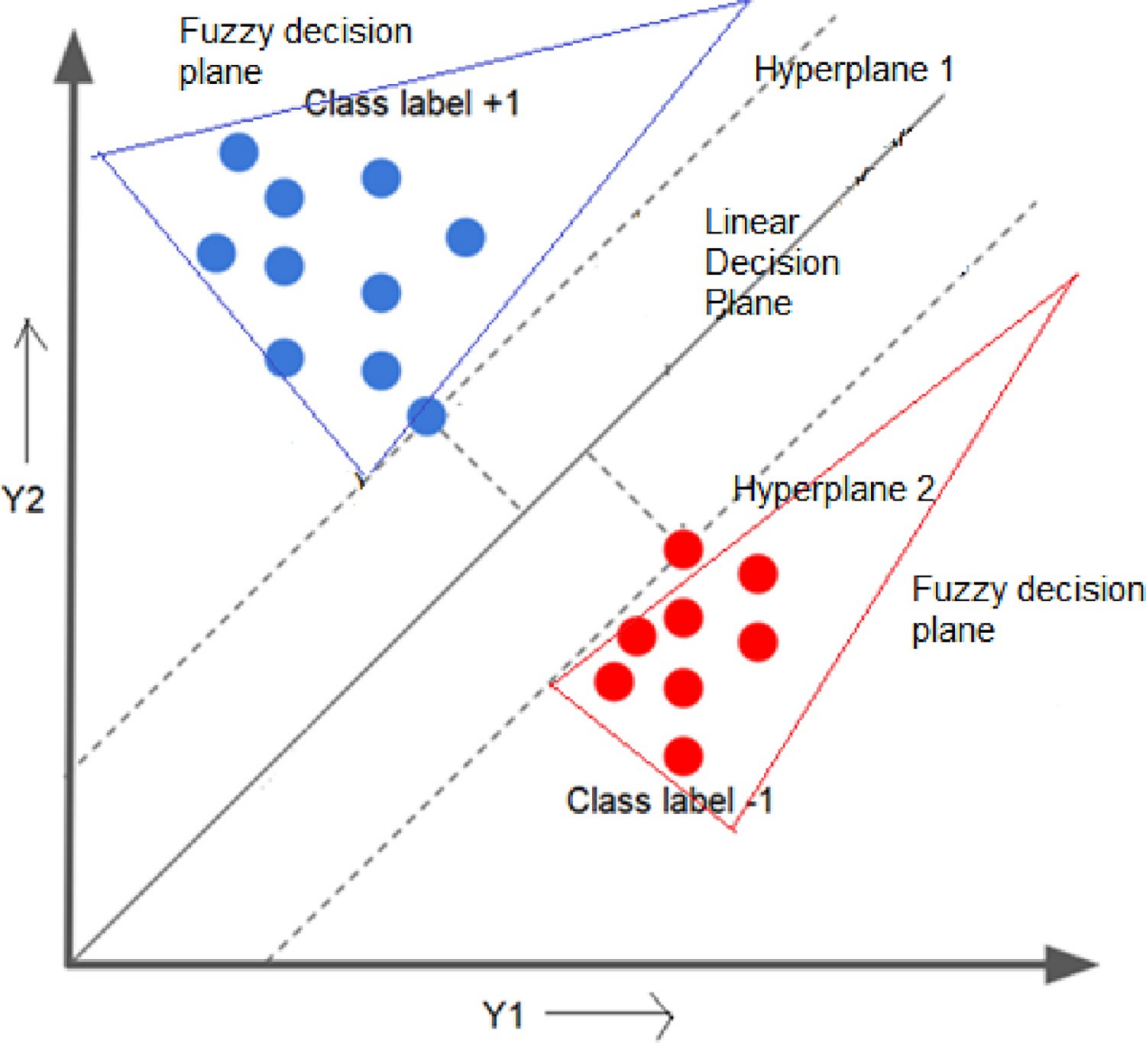
Fuzzy based TSVM hyperplane formation.

A novel VMD-ALO based deep learning fuzzy twin support vector machine model is devised in this research contribution to do superior prediction operation for the solar PV output power forecast. The classic variational model decomposition is employed over the solar PV farm datasets and the high intrinsic components gets extracted and these decomposed overcome the high fluctuations and provide the stable form of the data feature subsets. The stabilized form of the datasets are presented to the proposed DLFTSVM model, wherein the prediction is done by obtaining a fuzzy based twin hyperplanes that segregates the classes and carry out the prediction process. Fuzzy membership based TSVM is proposed with Gaussian Membership function to overcome the uncertainties in the hyperspace while formulating the hyperplanes. Fuzzy Gaussian membership function as well tunes the overall operation of the kernel functions of the twin SVM model. Hence, the hybrid deep learning based fuzzy TSVM algorithm proposed in this study combining the merits of the Gaussian membership function and twin support vector hyperplanes achieves most prominent hyperplanes to perform the PV output power prediction. Fig 3 illustrates the overall operation of the proposed VMD-ALO-DLFTSVM model. Fig 4 shows the twin decision hyperplanes attained with the fuzzy Gaussian membership function.

For the optimization problem defined in Eq (15), the two hyperplanes shall be formulated, but on considering the varied new data points the TSVM model is uncertain and the accuracy is not ascertained for the training solar datasets. The presence of inverse matrix operations and the multiplicand operator shows some critical complexity. Hence, this research study introduced the feature of fuzzy Gaussian membership function into the TSVM model and modelled new FTSVM with deep learning to determine most accurate twin support vector hyperplanes. In attaining the twin hyperplanes, the necessary parameters are assumed as fuzzy variables for the class labelled predicting data samples. Fuzzy membership function is defined and two fuzzy SVM decision planes are attained as shown in Fig 4.

**Fig 5:**
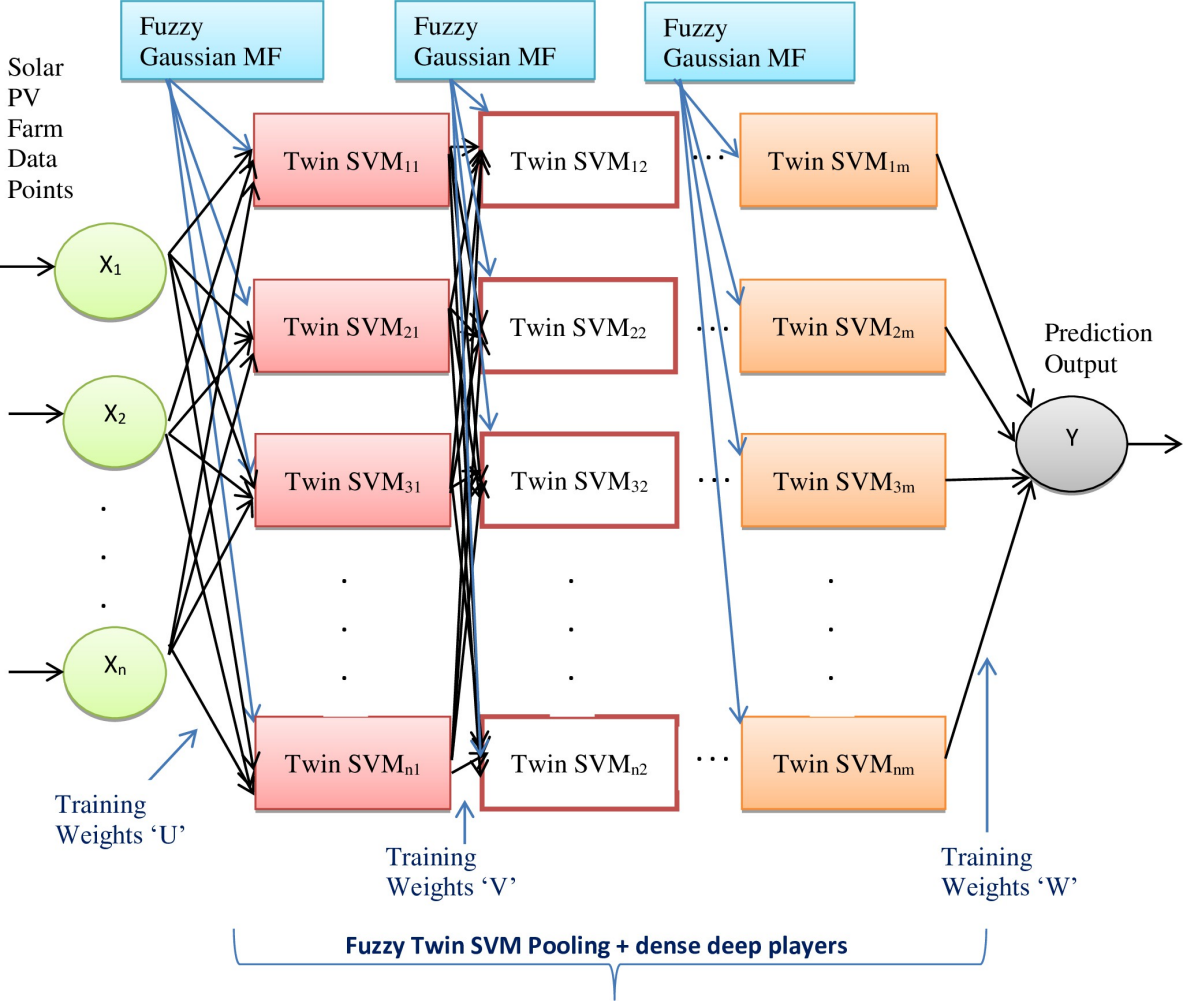
Architecture of proposed DLFTSVM solar PV power predictor.

For completely enclosing the spread of data points, the Gaussian basis function encloses the data points so that most appropriate hyperplane gets formed. As a result, DLFTSVM model enhances the prediction accuracy of the predictor by considering all the other data points that are far away from the hyperplane pertaining to a particular class. Fig 5 provides the architectural design of the DLFTSVM model.

For the proposed deep learning based FTSVM model, the quadratic optimization problem is defined as,

$$\begin{array}{l} \underset{w_{\left(\right)},w_{\left(1\right)},\delta_{\left(1\right)},\gamma_{1}}{\min}\frac{1}{2}{\left\|Pw_{\left(1\right)}+vw_{0(1)}\right\|}^{2}-u_{1}\gamma_{1}+\frac{1}{l_{2}}\mu_{2}^{T}\delta_{\left(2\right)}\;such\;that-(Qw_{\left(1\right)}+w_{0(1)})\geq \gamma_{1}-\delta_{\left(2\right)},\delta_{\left(2\right)}\geq 0,\gamma_{1}\geq 0\\ \underset{w_{(2},w_{\left(2\right)},\delta_{\left(2\right)},\gamma_{2}}{\min}\frac{1}{2}{\left\|Qw_{\left(2\right)}+vw_{0(2)}\right\|}^{2}-u_{2}\gamma_{2}+\frac{1}{l_{1}}\mu_{1}^{T}\delta_{\left(1\right)}\;such\;that-(Pw_{\left(2\right)}+w_{0(2)})\geq \gamma_{2}-\delta_{\left(1\right)},\delta_{\left(1\right)}\geq 0,\gamma_{2}\geq 0 \end{array}$$ (18)

In Eq (18), the regularization factors are *u*_*1*_ and *u*_*2*_, *μ*_*1*_ and *μ*_*2*_ denotes the fuzzy Gaussian membership function employed in this proposed model, *l*_*1*_ and *l*_*2*_ denotes the linear separability parameters. The main objective of the proposed DLFTSVM model is to determine two hyperplanes to perform the prediction mechanism. The optimization problem defined in Eq (18) can be solved to find solution using the Lagrange multiplier function,

$$\begin{array}{l} LM=\frac{1}{2}{\left\|Pw_{\left(1\right)}+vw_{0(1)}\right\|}^{2}-u_{1}\gamma_{1}+\frac{1}{l_{2}}\mu_{2}^{T}\delta_{\left(2\right)}+\\ \;\eta^{T}(Qw_{\left(1\right)}+w_{0(1)}+\gamma_{1}-\delta_{\left(2\right)})-\lambda^{T}\delta_{\left(2\right)}-\tau \gamma_{1} \end{array}$$ (19)

With the Lagrange multipliers *η*, *λ* and *τ* are greater than zero. Applying the Karush-Kuhn-Tucker conditions, Eq (18) gets transformed as,

$$\begin{array}{l} \underset{\eta }{\min}\frac{1}{2}\eta^{T}G{\left(H^{T}H\right)}^{-1}G^{T}\eta \;such\;that\;v\leq \eta \leq \frac{\mu_{2}}{l_{2}},v^{T}\eta \geq u_{1}\\ \underset{\lambda }{\min}\frac{1}{2}\lambda^{T}R{\left(S^{T}S\right)}^{-1}R^{T}\lambda \;such\;that\;v\leq \lambda \leq \frac{\mu_{1}}{l_{1}},v^{T}\lambda \geq u_{2} \end{array}$$ (20)

The solution to the defined problem with Lagrange multiplier of Eq (20) determines the hyperplanes based on the fuzzy membership functions *μ*_*1*_ and *μ*_*2*_. The modelled DLFTSVM is designed with the deep dense SVM layers and the pooling layers and the auto encoder and decoder units transforms all the input data points to low dimensional data components. Kernel functions are employed in the deep learning FTSVM to attain most suitable two hyperplanes for accurate prediction. The data non-linearity is handled by DL technique using,

$$\begin{array}{l} \mathrm{Encode}(z)=g_{\mathrm{encode}}(w_{0}+w_{y})\\ \mathrm{Decode}(z)=g_{\mathrm{decode}}(w_{0}+w_{y}^{T}) \end{array}$$ (21)

The encode vectors for all the deep fuzzy twin SVM layers are computed using,

$$\begin{array}{l} V_{encode\_1}=g_{encode\_1}(Z_{data\_pt})\\ V_{encode\_2}=g_{\mathrm{encode}\_2}(Z_{data\_pt})\\ V_{encode\_3}=g_{\mathrm{encode}\_3}(Z_{data\_pt})\\ \vdots \\ V_{encode\_n}=g_{\mathrm{encode}\_n}(Z_{data\_pt}) \end{array}$$ (22)

The final predicted output from the DLFTSVM predictor model becomes,

$$Y_{DL\_predicted}(Out)=g_{encode\_N+1}(Encode_{vector\_n})$$ (23)

In Eq (23), ‘*g*_*encode_N+1*_’ represents the trained entities of the deep FTSVM output layer and the new weights based on gradient evaluation is given by,

$$\begin{array}{l} w_{\mathrm{new}\_\mathrm{encode}}=W_{old\_encode}+\alpha_{lr}.\frac{\partial Error_{DL}}{\partial W_{new\_encode}}\\ w_{\mathrm{new}\_\mathrm{decode}}=w_{\mathrm{old}\_\mathrm{decode}}+\alpha_{lr}\frac{\partial Error_{DL}}{\partial W_{new\_decode}} \end{array}$$ (24)

The above procedure is carried out for the proposed DLFTSVM predictor model up to the error gets converged to a possible minimal value. Considering the computed output and the set target for the solar PV farm datasets, the error parameter is evaluated using,

$$E_{MSE}=\frac{1}{Max_{iter}}\sum_{i=1}^{Max_{iter}}(Y_{computed\_DLout}-Y_{set\_t\arg et}{)}^{2}$$ (25)

Table 2 provides the list of kernels employed during the training process of the new VMD_ALO-DLFTSVM predictor model. Fundamentally, seven kernel functions are most prominently employed. In this research study, based on the features of the kernel and their applicability, four kernel functions are employed in the DLFTSVM model to achieve better prediction accuracy by formulating the two decision hyperplanes. Laplace RBF kernel can handle the non-linearity in the data and helps to provide appropriate separate planes. The presence of cross-terms in the mathematical function shall be removed by the Bessel non-linear kernel function. As the solar PV farm data is of multi-dimensional, ANOVA RBF kernel has been chosen to attain the two hyperplanes. Hyperbolic tangential kernel is employed when higher variations in the data are present.

**Table 2:** Kernel functions adopted in the new DLFTSVM predictor.

Kernel functions	Functional definition of kernels adopted
Laplace RBF Kernel	$g_{\ker nel}(z_{p},z_{q})=\exp(-\frac{\left\|z_{p}-z_{q}\right\|}{\sigma })$
Bessel non-linear Kernel	$g_{\ker nel}(z_{p},z_{q})=(\frac{J_{v+1}(\sigma \|z_{p}-z_{q}\|)}{{\left\|z_{p}-z_{q}\right\|}^{-n(v+1)}})$, *J*–Bessel function of first kind
ANOVA RBF Kernel	$g_{\ker nel}(z_{p},z_{q})=\sum_{i=1}^{q}\exp{\left(-\sigma {\left(z_{p}^{i}-z_{q}^{i}\right)}^{2}\right)}^{d}$, *d*–degree of polynomial
Hyperbolic tangential Kernel	$g_{\ker nel}(z_{p},z_{q})=\tanh(kz_{p}.z_{q}+\rho )\;s.t\;k>0\;and\;\rho <0$

### Benchmark solar power generation datasets

The solar power generation datasets employed in this research study pertains to the two solar 250 MW PV farms in India–Plant 1 at Gandikotta, Andhra Pradesh and Plant 2 at Nasik, Maharashtra collected over duration of 34 day period during May-June 2020. Both the plants are 50MW capacity and their yield is dependent on irradiation. Apart from the regular temperature, the irradiation and ambient temperature rise shoots up and after a threshold limit, the yield increases. The observations are recorded for both plants in a span of 15 minute intervals. The valid Daily_Yield, Ambient_Tempertaure, Irradiation and Total_Yield are recorded and these are employed as the input variables to the proposed VMD-ALO-DLFTSVM approach. The output variable is the predicted total yield of the solar power. The total yield will be the total yield of the inverter till that particular point of time. Table 3 provides the sample of data for both the plants pertaining to the solar PV output power generation [79].

**Table 3:** Sample of solar PV power generation dataset.

Daily_Yield(W)	Ambient_Temperature	Irradiation	Total_Yield(W)
1391.571429	26.43078207	0.405348573	6340771.571
970.4285714	26.8318298	0.312426795	7117121.429
1307.571429	27.6209698	0.623152649	6260866.571
1542.625	27.98836207	0.344884036	6185187.625
1509	27.51672787	0.2492484	6989268
1471.142857	27.45010767	0.541205977	7604431.143
1596.125	28.63219187	0.670675372	7160560.125
1471.285714	28.76891273	0.572283477	7207879.286
1466.428571	29.3514426	0.455148383	7030139.429
1541.571429	28.85470787	0.361961654	7181507.571
1494.857143	29.41081933	0.636560881	6523666.857
1503.285714	30.21606229	0.585787214	7099602.286
1542	30.2870728	0.557069383	6272897
1311.142857	30.81104933	0.467986869	6318114.143
1572.75	31.30537507	0.514962587	7179564.75
1529.5	31.50729773	0.787866029	6186695.25
1580.125	32.14768473	0.649247629	7170682.125
1572	32.3914204	0.761243312	7113065
1574.75	32.62279607	0.416035101	7018406.75
1584.375	32.49706447	0.489243946	7040265.375
1535.25	32.5246214	0.574561224	6784133.25
1558	32.67847087	0.560985624	7009424
1551.75	33.7631854	0.735083463	6340931.75
1158	34.13076993	0.893661491	7117309
1440	34.08138427	0.466788824	6260999
1723.142857	33.69572221	0.542138496	6185368.143
1661.857143	33.89057607	0.398888543	6989420.857

## Results

The novel VMD-ALO-DLFTSVM predictor model developed in this research study is validated and tested for its superiority for solar PV output prediction for the two solar farmdatasets and the performance metrics are evaluated. The complete simulation process of the prediction model is carried out in MATLAB R2021a environment on an Intel dual core i5 processor of 8GB physical memory. Initially, for the raw data variational mode decomposition is applied and based on the intrinsic frequency, the data are decomposed and presented as input to the DLFTSVM predictor model. The classic ALO algorithm is invoked after the first run of the predictive algorithm and the weight and bias parameters of the DLFTSVM are tuned for their optimality and then the deep learning progresses. With the data decomposed from VMD module and optimal parameters attained from the ALO tuning, the deep learning intends to determine the most nearer predictive value for the solar PV output power.

For evaluating the developed predictor model, the metrics computed during the progress of deep learning are Mean Absolute Error (MAE), Mean Square Error (MSE), Root Mean Square Error (RMSE) and Prediction Accuracy (P_Acc_) and they are defined by the following equation,

$$\begin{array}{l} MAE=\frac{1}{N}\sum_{j=1}^{N}\left|Y_{predicted\_j}-Y_{original\_j}\right|\\ MSE=\frac{1}{N}{\sum_{j=1}^{N}\left(Y_{predicted\_j}-Y_{original\_j}\right)}^{2}\\ RMSE=\sqrt{\frac{1}{N}{\sum_{j=1}^{N}\left(Y_{predicted\_j}-Y_{original\_j}\right)}^{2}}\\ P_{Acc}=\frac{1}{N}\sum_{j=1}^{N}O_{j},O_{j}=\{\begin{array}{l} 1,\;if(Y_{predicted\_j+1}-Y_{original\_j})(Y_{original\_j+1}-Y_{original\_j})>0\\ 0,\;Otherwise \end{array} \end{array}$$ (26)

**Fig 6:**
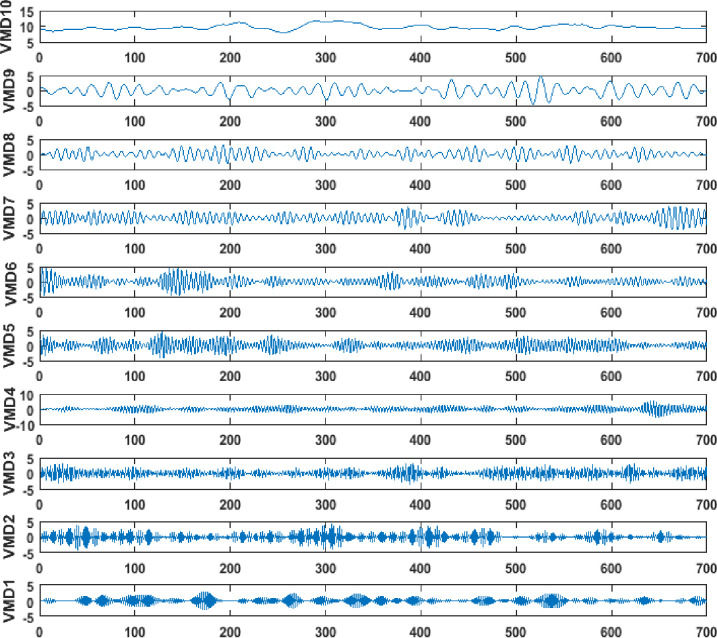
VMD output for the considered solar PV farm.

In Eq (26), the number of data samples is specified with ‘*N*’, ‘*Y*_*original*_’ indicates the actual solar farm data and ‘*Y*_*predicted*_’ specifies the predicted output. Table 4 provides the simulation parameters of the proposed predictor model. Fig 6 provides the VMD output of the considered solar data samples.

**Table 4:** Simulation parameters of the proposed predictor model.

Parameters	Parametric Values
Number of ants	40
Intrinsic mode frequencies	10
Learning rate	0.2
Structure of DL model	4-4-3-4-1
Maximum iterations	Till the convergence is reached
Convergence criterion	10^−6^
Convergence cost function	$E_{MSE}=\frac{1}{Max_{iter}}\sum_{i=1}^{Max_{iter}}(Y_{computed\_DLout}-Y_{set\_t\arg et}{)}^{2}$
Deep learning rule	Gradient Descent technique
Fuzzy membership function	Gaussian membership function
Control parameter τ	0.03
Batch size	40 samples
Trial runs	36

The decomposed signals in respect of the solar PV farm data are fed into the designed deep learning based fuzzy twin support vector machine model. The deep FTSVM is designed with input layer of 4 neurons (daily yield, ambient temperature, irradiation, and total yield), three deep dense layers with 4-3-4 neuronal structure and one output layer with single output neuron for total yield prediction. The weights and the bias are initially set to small random values and during the progressive deep learning training, the weights and bias are optimally tuned with the ALO algorithmic flow. The weights and bias will form the number of ants to be generated and the attainment of minimal MSE value tends to be the convergence for the algorithm for the considered solar PV plant 1 and plant 2 datasets.

**Fig 7:**
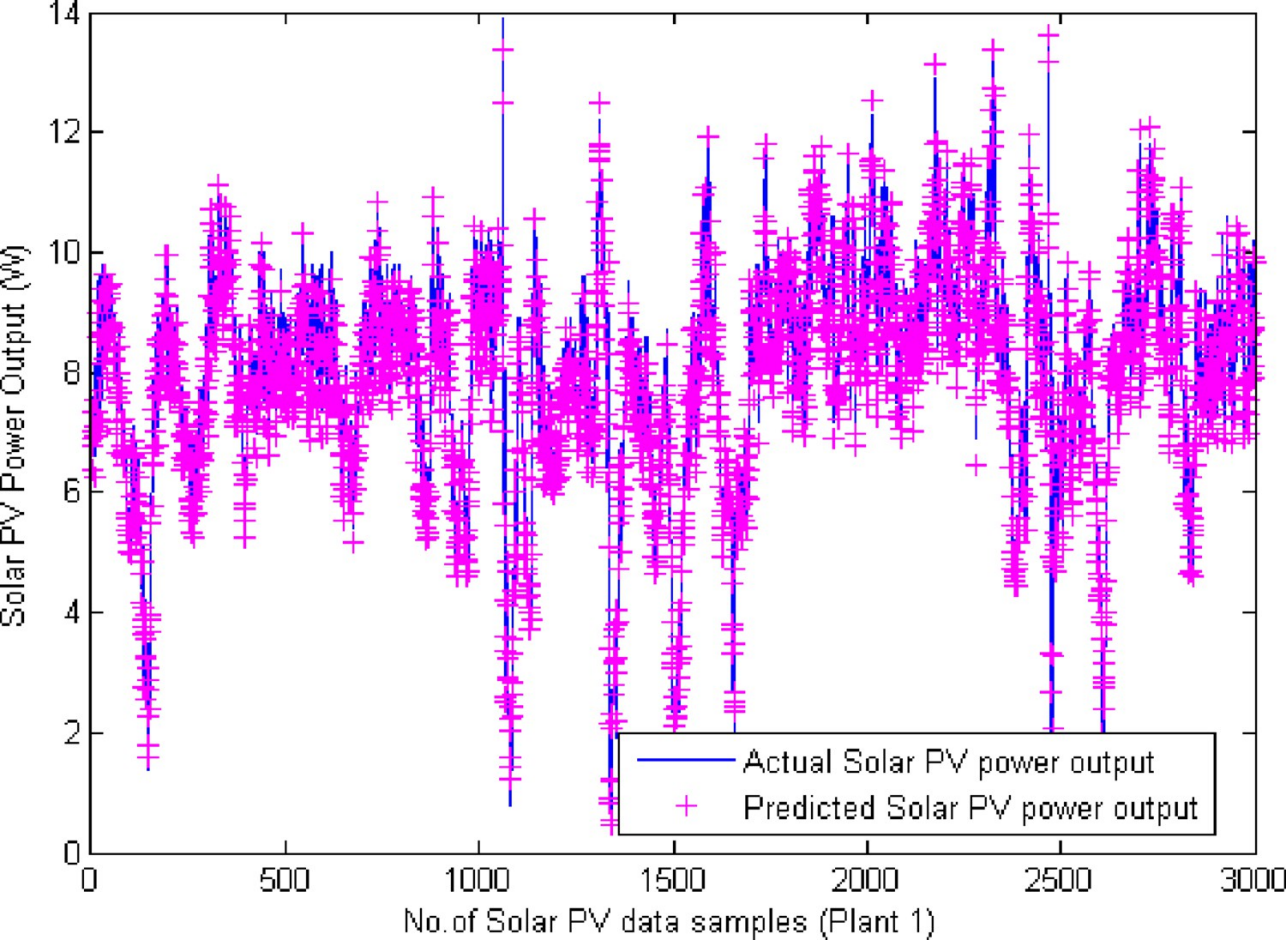
Plot of actual and predicted solar PV output power (Plant 1 solar PV farm).

**Fig 8:**
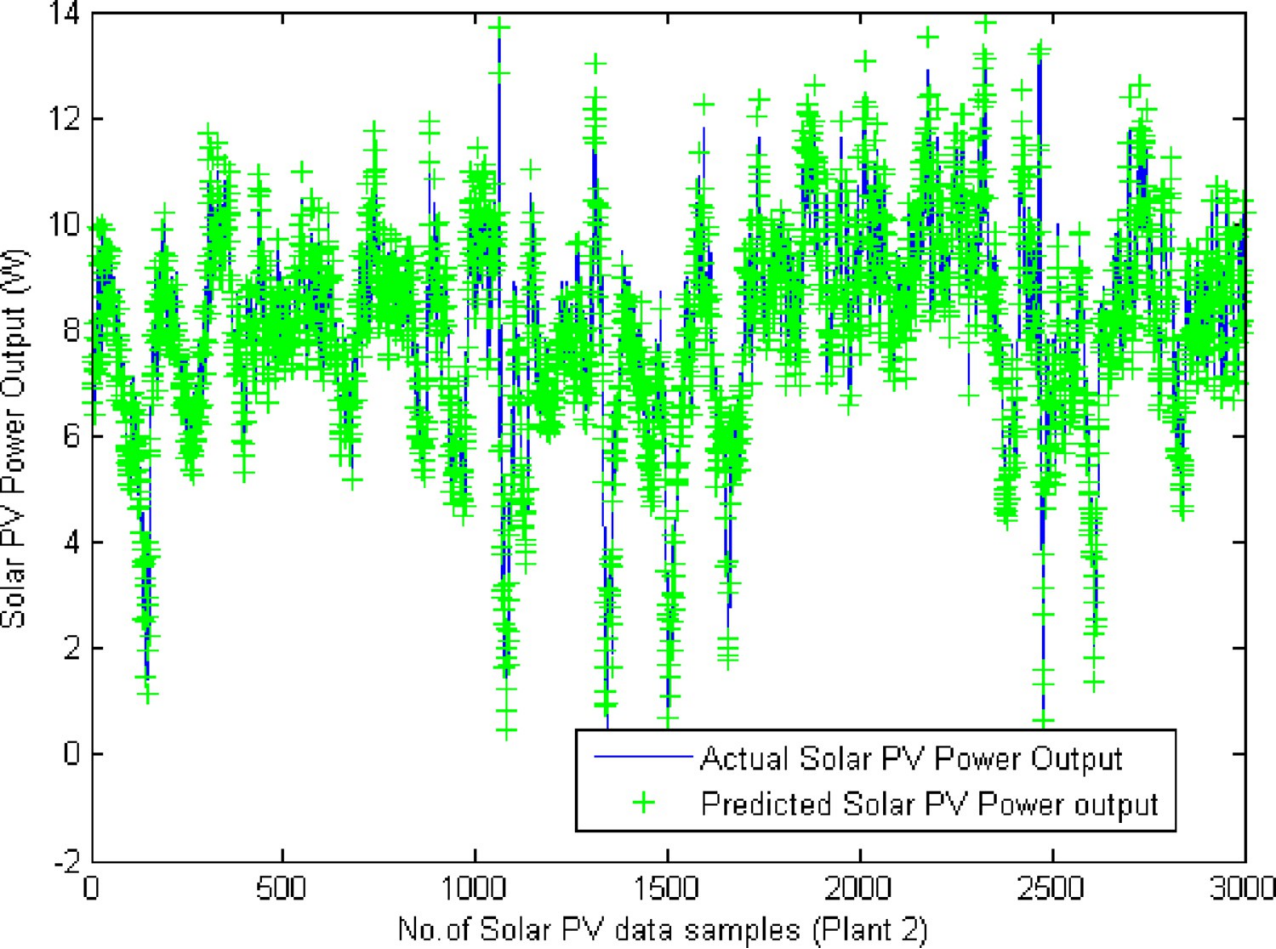
Plot of actual and predicted solar PV output power (Plant 2 solar PV farm).

On carrying out the simulation process for both the datasets, the predicted PV output power is evaluated based on the presented input values and the *MAE*, *MSE*, *RMSE* and *P*_*Acc*_ are computed and tabulated in Table 5. Figs 7 and 8 illustrates the simulated plots of the predicted output power with that of the original total yield output power. It is lucid from the plots that the predicted output solar power is in par with the actual data for both the plants confirming the efficacy of the proposed predictor model. Out of the four kernels employed to performing the prediction with the two hyperplane formation, the ANOVA RBF kernel has resulted in better values of the performance metrics than the other kernels. This is due to the ability of ANOVA radial basis function kernel to handle multi-dimensional data and to thereby formulate the hyperplanes.

**Table 5:** Evaluated performance metrics using VMD-ALO-DLFTSVM predictor.

Solar PV Farm	Kernel Functions	Performance Metrics			
		MAE	MSE	RMSE	PAcc
Plant 1 Solar PV Farm	Laplace RBF Kernel	1.2769	0.015481	0.12447	0.6475
	Bessel non-linear Kernel	0.5418	5.3149×10^−4^	0.02305	0.8114
	ANOVA RBF Kernel	**0.2217**	**9.3507×10** ^**−6**^	**0.00306**	**0.9564**
	Hyperbolic tangential Kernel	1.0013	0.003647	0.06039	0.7166
Plant 2 Solar PV Farm	Laplace RBF Kernel	1.9276	0.014670	0.121119	0.6943
	Bessel non-linear Kernel	0.4472	0.000548	0.023409	0.8847
	ANOVA RBF Kernel	**0.0918**	**3.1492×10** ^**−5**^	**0.00845**	**0.9146**
	Hyperbolic tangential Kernel	1.1247	0.002491	0.04991	0.7519

**Fig 9:**
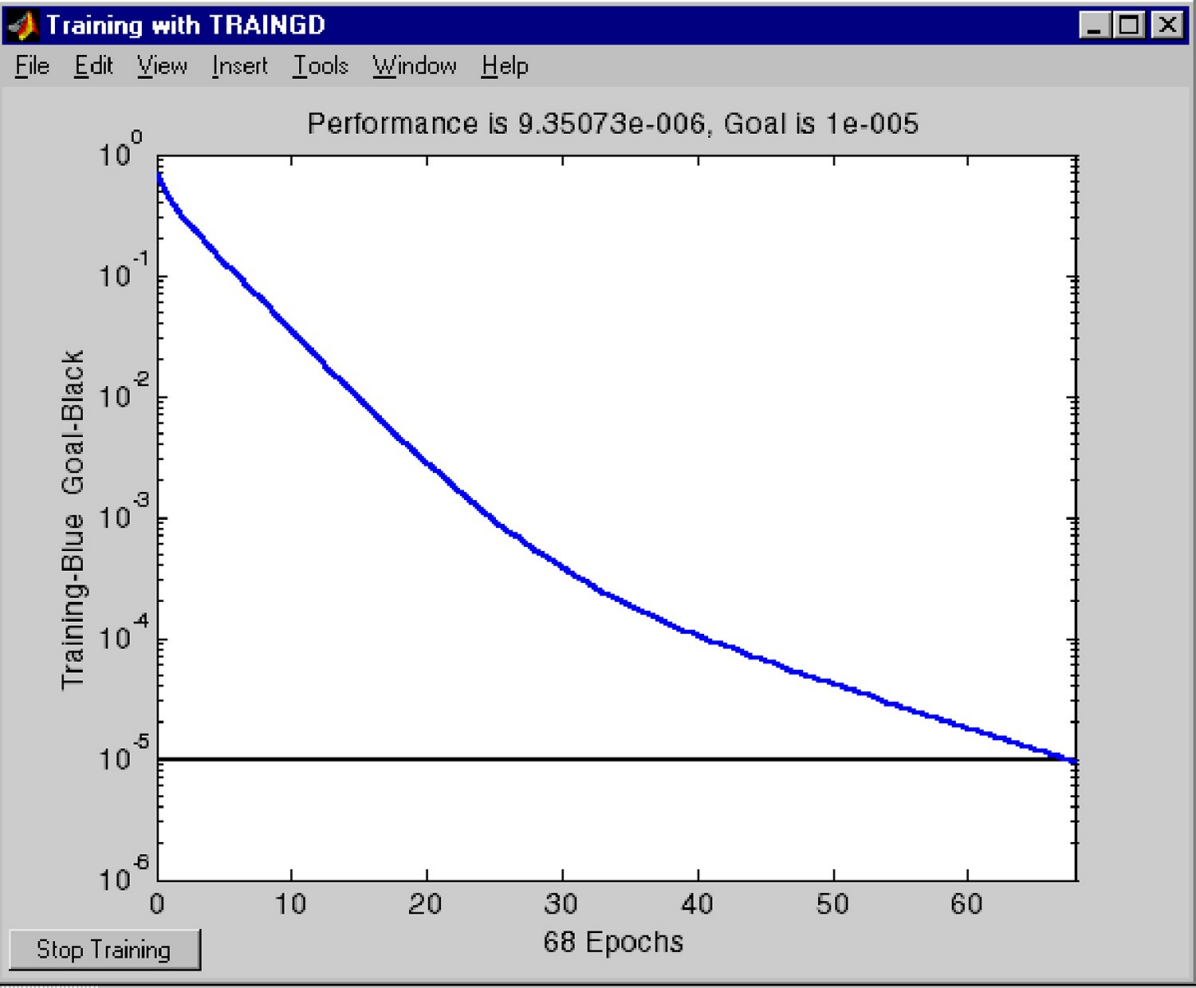
Convergence plot for the proposed predictor model during DL training.

Table 5 gives the performance metrics evaluated for varied kernel functions using the proposed VMD-ALO-DLFTSVM predictor model. Table 5 confirms the attainment of minimal error values for MAE, MSE and RMSE and higher values of the prediction accuracy. With respect to all kernels, ANOVA RBF kernel has computed values of 0.2217, 9.3507×10^−6^, 0.00306 and 0.9564 for *MAE*, *MSE*, *RMSE* and *P*_*Acc*_ respectively for Plant 1 respectively and for plant 2 solar PV farm using the proposed model the computed values of *MAE*, *MSE*, *RMSE* and *P*_*Acc*_ are 0.0918, 3.1492×10^−5^, 0.00845, 0.9146 respectively. The evaluated MSE values with respect to the number of iterations elapsed during training process is given in Table 6 for both the solar PV farms. MSE value of 9.3507×10^−6^ is elapsed at 68^th^ epoch for training process and during testing process, the MSE was 3.1492×10^−5^ at 76^th^ epoch for testing process. Fig 9 provides the convergence plot of the proposed predictor model during deep learning process. Table 7 presents the sample of predicted solar PV output power compared with the original solar PV output for plant 1 and plant 2. The predicted values confirm that they are near equal to that of the original solar PV power output for the solar PV plants considered for analysis.

**Table 6:** MSE evaluated over iterations for DLFTSVM predictor.

Plant 1 Solar PV Datasets		Plant 2 Solar PV Datasets	
Iterations	Mean Square Error	Iterations	Mean Square Error
10	0.9651	10	1.2479
20	4.5671×10^−3^	20	0.9934
30	5.0037×10^−4^	30	7.2261×10^−3^
40	1.1249×10^−4^	40	8.0093×10^−4^
50	6.2127×10^−5^	50	6.2371×10^−4^
60	1.2267×10^−5^	60	1.1672×10^−4^
**68**	**9.3507×10** ^**−6**^	70	9.6651×10^−5^
At the 68^th^ Iteration, it has reached the convergence and attained the minimal MSE value		76	**3.1492×10** ^**−5**^
		At the 76^th^ Iteration, it has reached the convergence and attained the minimal MSE value	

**Table 7:** Sample predicted output samples with VMD-ALO-DLFTSVM predictor.

Plant 1		Plant 2	
Original Total Output Yield (W)	Predicted Total Output Yield (W)	Original Total Output Yield (W)	Predicted Total Output Yield (W)
6259559	6258900	1708083348	1708090000
6183645	6201489	339923	340163
6987759	6987611	120964108	121065731
7602960	7601995	2211962	2211995
7158964	7159003	106656621	106657014
7206408	7206399	209143593	209155640
7028673	7028596	2429011	2430017
6522172	6520018	1215278736	1215279110
7098099	7100974	2247719577	2247720137
6271355	6271255	1704250	1704727
6316803	6317120	19941526	19940961
7177992	7175663	1794958634	1794958516
6185184	6189451	282592810	282592564
7169102	7170023	2453646	2453410
7111493	7111556	111512591	111512761
7016832	7017238	1348350801	1348349967
7038681	7038690	838421377	838420965
6782598	6782614	329509085	329505514
7158964	7159741	1412083119	1412079664
7206408	7206561	181695261	181695199
7028673	7029410	593580025	593580174

## Discussion

The merits of the proposed VMD-ALO-DLFTSVM predictor model lies in its capability to formulate the most prominent two hyperplanes using the fuzzy Gaussian membership function and that of the kernel functions in the multi-dimensional dataset hyperspace. Additionally, the basic ALO algorithm tends to achieve the optimal value of weights ad bias metrics for the deep learning fuzzy twin SVM model. As a result of optimized weight and bias values, the existence of local and global optima is overcome. The architecture of the deep learning based FTSVM model has achieved better prediction accuracy by avoiding the under fitting and over fitting occurrences. For the plant 1 and plant 2 datasets, 5-fold cross validation is employed to carry out the simulation process and the predicted output values are computed. VMD facilitates in protecting the information of the datasets based on the intrinsic frequency components and loss of information gets protected. This intends to provide the most accurate solar PV data with noise removal to the next stage of the ALO optimization technique and the deep learning technique. The fuzzy model generates the membership functions so that it handles the complexity and intends to increase the prediction accuracy. With the deep hidden dense layers, suitable predicting hyper plane gets formulated and the effectiveness of suggested technique is established. It overcomes the local and global problems and stagnation issues with appropriate scalability.

The limitations of the suggested technique are the increased computational complexity of the model and the randomness during the initial training of the algorithm. Also, at times premature convergence was noticed, but this was overcome by the optimized weight and other parameters evaluated during the run of the ALO algorithmic process.

### Comparative analysis

The predictor model developed in this study forecasted the solar PV power output; that is the total yield of the solar plants was evaluated based on the daily yield, ambient temperature, irradiation and total yield. For the two considered solar PV farms, the VMD-ALO-DLFTSVM model intended to attained better prediction accuracy and minimized value of mean square error during the training and testing process. Table 8 presents a comparative analysis of the proposed predictor with that of the prediction techniques from previous works [18, 27, 29, 33, 40, 61]. For all these previous methods, the same datasets were presented as input and their MSE and prediction accuracy was attained. It is well elucidated from Table 8, that the proposed VMD-ALO based deep learning FTSVM predictor model with MSE of 9.3507×10^−6^ and prediction accuracy of 0.9564 has proved to be better than other techniques for the solar PV plant 1 dataset. In respect of solar PV plant 2 dataset, the new predictor model attained MSE of 3.1492×10^−5^ and prediction accuracy of 0.9146 comparatively better than previous predictors proving its superiority. The signal decomposition based on the intrinsic frequency and the applicability of ant lion optimizer to attain optimal weights for the training of deep learning model has achieved better predicted solar PV power output in par with that of the original PV power output for both the solar PV datasets.

**Table 8:** Comparisons of proposed technique with other techniques.

Prediction Techniques Adopted	Solar PV Plant 1		Solar PV Plant 2	
	MSE Value	Prediction Accuracy	MSE Value	Prediction Accuracy
SVM approach	7.2196	0.3148	10.3247	0.5017
Fuzzy SVM approach	7.0027	0.5589	8.4127	0.5584
Twin SVM model	4.1028	0.6134	6.0349	0.5981
Fuzzy Twin SVM model	3.6629	0.6647	4.1247	0.6014
PSO-BP neural model [18]	1.4218	0.6981	4.0029	0.6543
GA–BP neural model [18]	1.0092	0.7754	3.1473	0.7291
ANN model [33]	0.9280	0.7842	1.1420	0.7754
SVM big data model [61]	0.2217	0.8046	0.8149	0.7963
RNN–LSTM model [27]	0.1627	0.8172	0.3651	0.8296
AMPSO-CLSTM neural model [29]	0.1129	0.8845	0.1024	0.8541
LSTM-ARMA combined model [40]	0.0916	0.9014	0.0981	0.8837
MFA-Elman technique [3]	0.0887	0.9247	0.09116	0.8965
Wavelet+Deep learning neural model [11]	0.0859	0.9291	0.08875	0.8987
Ensemble neural technique [14]	0.0548	0.9306	0.00629	0.9005
**Proposed VMD-ALO-DLFTSVM predictor**	**9.3507×10** ^**−6**^	**0.9564**	**3.1492×10** ^**−5**^	**0.9146**

**Fig 10:**
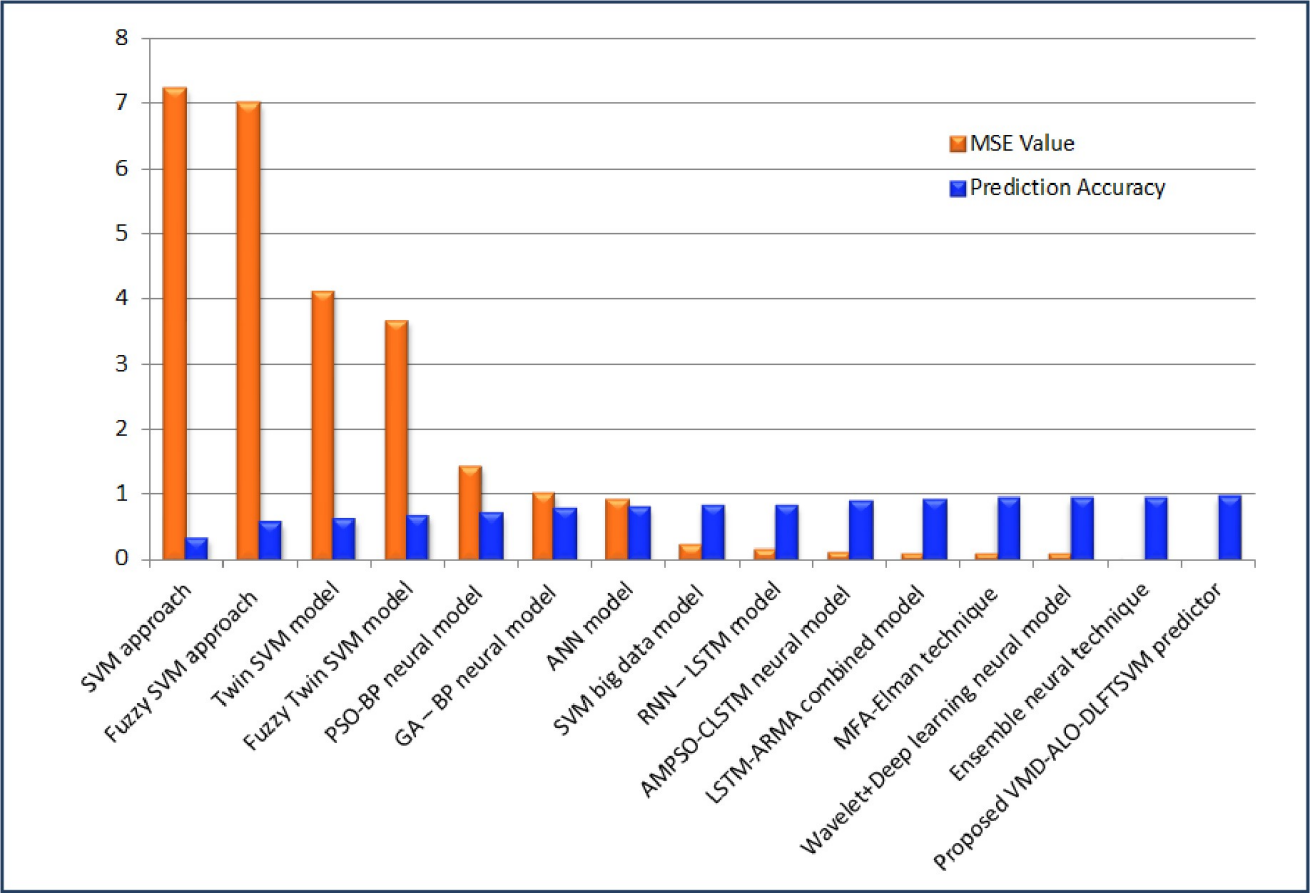
Plot for comparisons MSE value and accuracy of proposed technique with other techniques (Solar PV plant 1).

**Fig 11:**
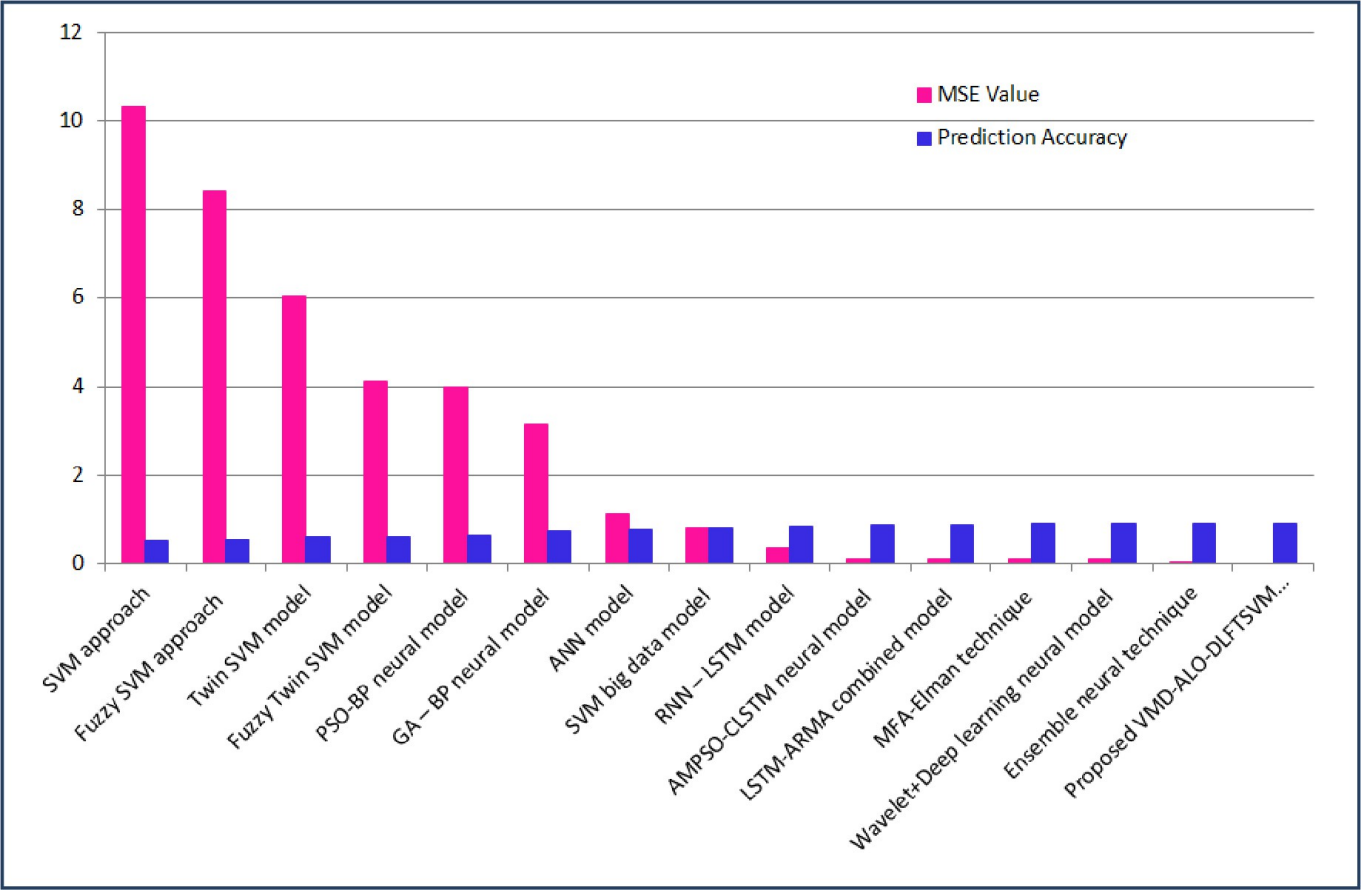
Plot for comparisons of MSE value and accuracy of proposed technique with other techniques (Solar PV plant 2).

Figs 10 and 11 provides the comparison plot of the proposed technique over the traditional and other new methods in respect of the mean square error and prediction accuracy for solar PV power plant 1 and power plant 2 datasets. For solar PV plant 1 the mean square error using twin SVM model is 4.1028, for PSO-BP neural model it is 1.4218, GA-BP neural model is 1.0092, using LSTM model it is 0.0916 and for the proposed VMD-ALO-DLFTSVM predictor it is 9.3507×10^−6^ and in respect of accuracy it is 95.64%, which is higher compared with 31.48%, 55.89%, 61.34%, 88.45% and 93.06% for SVM, Fuzzy SVM, Twin SVM, AMPSO-LSTM and Ensemble model, proving the effectiveness of proposed technique. Considering the evaluated results for the solar PV Plant 2, the MSE reduced from 10.3247 for SVM to a most minimal value of 3.1492×10^−5^ using the suggested technique. Also, the accuracy increased from 50.17% for SVM, 55.84% for Fuzzy SVM, 59.81% for Twin SVM, 82.96% for RNN-LSTM, 90.05% for Ensemble neural model to 91.46% using the proposed predictor model. The values claim the effectiveness of the proposed model over the other traditional and new methods available in the previous studies.

## Conclusions

The prediction of solar PV power output for 250MW solar farms has been carried out in this research study by developing a novel variational mode decomposition–ant lion optimizer based deep learning fuzzy twin support vector machine model. The proposed predictor model performed the forecasting of the solar power output by formulating two hyperplanes. The process to achieve the most optimal hyperplanes for prediction was carried out by the deep learning with its weights optimized using the ant lion optimizer algorithm. The new VMD-ALO-FTSVM predictor has resulted in better prediction accuracy and minimal mean square error than the other techniques considered for comparison from previous works. The predicted solar power output has observed to be near equal to that of the original solar PV farm data substantiating the superiority than earlier predictive models.

## References

[1] Etxegarai G, López A, Aginako N, Rodríguez F. An analysis of different deep learning neural networks for intra-hour solar irradiation forecasting to compute solar photovoltaic generators’ energy production. Energy for Sustainable Development. 2022;68 (6):1–7.

[2] Markovics D, Mayer MJ. Comparison of machine learning methods for photovoltaic power forecasting based on numerical weather prediction. Renewable and Sustainable Energy Reviews. 2022;161 (3):112364. doi: 10.1016/j.rser.2022.112364

[3] Ma X, Zhang X. A short-term prediction model to forecast power of photovoltaic based on MFA-Elman. Energy Reports. 2022;8 (7):495–507.

[4] Agga A, Abbou A, Labbadi M, El Houm Y, Ali IH. CNN-LSTM: An efficient hybrid deep learning architecture for predicting short-term photovoltaic power production. Electric Power Systems Research. 2022;208 (4):107908. doi: 10.1016/j.epsr.2022.107908

[5] Zhang H, Shi J, Zhang C. A hybrid ensembled double-input-fuzzy-modules based precise prediction of PV power generation. Energy Reports. 2022;8 (7):1610–21.

[6] Li J, Liu Q. Forecasting of short-term photovoltaic power generation using combined interval type-2 Takagi-Sugeno-Kang fuzzy systems. International Journal of Electrical Power & Energy Systems. 2022;140 (9):108002.

[7] Li J, Niu H, Meng F, Li R. Prediction of Short-Term Photovoltaic Power Via Self-Attention-Based Deep Learning Approach. Journal of Energy Resources Technology. 2022;144(10):101301.

[8] Son Y, Yoon Y, Cho J, Choi S. Cloud Cover Forecast Based on Correlation Analysis on Satellite Images for Short-Term Photovoltaic Power Forecasting. Sustainability. 2022;14 (8):4427.

[9] Wang J, Zhou Y, Li Z. Hour-ahead photovoltaic generation forecasting method based on machine learning and multi objective optimization algorithm. Applied Energy. 2022;312 (4):118725.

[10] Ahmed R, Sreeram V, Togneri R, Datta A, Arif MD. Computationally expedient Photovoltaic power Forecasting: A LSTM ensemble method augmented with adaptive weighting and data segmentation technique. Energy Conversion and Management. 2022;258 (1):115563.

[11] Rodríguez F, Azcárate I, Vadillo J, Galarza A. Forecasting intra-hour solar photovoltaic energy by assembling wavelet based time-frequency analysis with deep learning neural networks. International Journal of Electrical Power & Energy Systems. 2022;137 (5):107777.

[12] Huang X, Li Q, Tai Y, Chen Z, Liu J, Shi J, et al. Time series forecasting for hourly photovoltaic power using conditional generative adversarial network and Bi-LSTM. Energy. 2022;246 (5):123403.

[13] Mas’ ud AA. Comparison of three machine learning models for the prediction of hourly PV output power in Saudi Arabia. Ain Shams Engineering Journal. 2022;13(4):101648.

[14] Nespoli A, Leva S, Mussetta M, Ogliari EG. A Selective Ensemble Approach for Accuracy Improvement and Computational Load Reduction in ANN-Based PV Power Forecasting. IEEE Access. 2022;10:32900–11.

[15] Elsaraiti M, Merabet A. Solar power forecasting using deep learning techniques. IEEE Access. 2022;10 (3):31692–8.

[16] Mughal SN, Sood YR, Jarial RK. Design and optimization of photovoltaic system with a week ahead power forecast using autoregressive artificial neural networks. Materials Today: Proceedings. 2022;52 (1):834–41.

[17] Ofori-Ntow Jnr E, Ziggah YY, Rodrigues MJ, Relvas S. A New Long-Term Photovoltaic Power Forecasting Model Based on Stacking Generalization Methodology. Natural Resources Research. 2022:1–23.

[18] Li Y, Zhou L, Gao P, Yang B, Han Y, Lian C. Short-term power generation forecasting of photovoltaic plant based on PSO-BP and GA-BP neural networks. Frontiers in Energy Research:958.

[19] Huang Y, Zhou M, Yang X. Ultra-short-term photovoltaic power forecasting of multifeature based on hybrid deep learning. International Journal of Energy Research. 2022;46(2):1370–86.

[20] Akhter MN, Mekhilef S, Mokhlis H, Ali R, Usama M, Muhammad MA, et al. A hybrid deep learning method for an hour ahead power output forecasting of three different photovoltaic systems. Applied Energy. 2022;307 (2):118185.

[21] Ardila VM, Maciel JN, Ledesma JJ, Junior OH. Fuzzy Time Series Methods Applied to (In) Direct Short-Term Photovoltaic Power Forecasting. Energies. 2022;15(3):845.

[22] Carneiro TC, de Carvalho PC, Alves dos Santos H, Lima MA, Braga AP. Review on photovoltaic power and solar resource forecasting: current status and trends. Journal of Solar Energy Engineering. 2022 Feb 1;144(1): 010801

[23] Zhang N, Ren Q, Liu G, Guo L, Li J. Short-term PV Output Power Forecasting Based on CEEMDAN-AE-GRU. Journal of Electrical Engineering & Technology. 2022: (1):1182–1194

[24] Pretto S, Ogliari E, Niccolai A, Nespoli A. A New Probabilistic Ensemble Method for an Enhanced Day-Ahead PV Power Forecast. IEEE Journal of Photovoltaics. 2022.: 12(2):581–588.

[25] Beigi M, Beigi Harchegani H, Torki M, Kaveh M, Szymanek M, Khalife E, et al. Forecasting of Power Output of a PVPS Based on Meteorological Data Using RNN Approaches. Sustainability. 2022;14(5):3104.

[26] Elizabeth Michael N, Mishra M, Hasan S, Al-Durra A. Short-term solar power predicting model based on multi-step CNN stacked LSTM technique. Energies. 2022;15(6):2150.

[27] Akhter MN, Mekhilef S, Mokhlis H, Almohaimeed ZM, Muhammad MA, Khairuddin AS, et al. An Hour-Ahead PV Power Forecasting Method Based on an RNN-LSTM Model for Three Different PV Plants. Energies. 2022;15(6):2243.

[28] Zhang W, Li Q, He Q. Application of machine learning methods in photovoltaic output power prediction: A review. Journal of Renewable and Sustainable Energy. 2022;14(2):022701.

[29] Yu S, Zheng Y, Han R, Gong C. An integrated AMPSO-CLSTM model for photovoltaic power generation prediction. Frontiers in Energy Research. 2022 3:264.

[30] Ibrahim IM, Belanger J, Shehata AS, Shehata AI, Davol A. Enhancement of photovoltaic power farms using a new power prediction approach. International Journal of Energy Research. 2022;46(4):4222–46.

[31] Simeunovic J, Schubnel B, Alet PJ, Carrillo RE. Spatio-temporal graph neural networks for multi-site PV power forecasting. IEEE Transactions on Sustainable Energy. 2021; 13(2):1210–1220.

[32] Zazoum B. Solar photovoltaic power prediction using different machine learning methods. Energy Reports. 2022;8 (4):19–25.

[33] Geetha A, Santhakumar J, Sundaram KM, Usha S, Thentral TT, Boopathi CS, et al. Prediction of hourly solar radiation in Tamil Nadu using ANN model with different learning algorithms. Energy Reports. 2022;8 (2):664–71.

[34] Lopes S, Cari E, Hajimirza S. A Comparative analysis of Artificial Neural Networks for Photovoltaic Power Forecast using remotes and local measurements. Journal of Solar Energy Engineering. 2022;144(2): 021007

[35] Wentz VH, Maciel JN, Gimenez Ledesma JJ, Ando Junior OH. Solar Irradiance Forecasting to Short-Term PV Power: Accuracy Comparison of ANN and LSTM Models. Energies. 2022;15(7):2457.

[36] An Y, Dang K, Shi X, Jia R, Zhang K, Huang Q. A Probabilistic Ensemble Prediction Method for PV Power in the Nonstationary Period. Energies. 2021;14(4):859.

[37] Lee D, Jeong JW, Choi G. Short Term Prediction of PV Power Output Generation Using Hierarchical Probabilistic Model. Energies. 2021;14(10):2822.

[38] Wang M, Peng J, Luo Y, Shen Z, Yang H. Comparison of different simplistic prediction models for forecasting PV power output: Assessment with experimental measurements. Energy. 2021;224 (6):120162.

[39] Wang L, Shi J. A Comprehensive Application of Machine Learning Techniques for Short-Term Solar Radiation Prediction. Applied Sciences. 2021;11(13):5808.

[40] Jiang Y, Zheng L, Ding X. Ultra-short-term prediction of photovoltaic output based on an LSTM-ARMA combined model driven by EEMD. Journal of Renewable and Sustainable Energy. 2021;13(4):046103.

[41] Abedinia O, Bagheri M, Agelidis VG. Application of an adaptive Bayesian-based model for probabilistic and deterministic PV forecasting. IET Renewable Power generation. 2021; 15 (12), 2699–2714

[42] Zhao Z, Chen K, Chen Y, Dai Y, Liu Z, Zhao K, et al. An Ultra-Fast Power Prediction Method Based on Simplified LSSVM Hyperparameters Optimization for PV Power Smoothing. Energies. 2021;14(18):5752.

[43] Qu J, Qian Z, Pei Y. Day-ahead hourly photovoltaic power forecasting using attention-based CNN-LSTM neural network embedded with multiple relevant and target variables prediction pattern. Energy. 2021;232 (10):120996.

[44] Mohana M, Saidi AS, Alelyani S, Alshayeb MJ, Basha S, Anqi AE. Small-Scale Solar Photovoltaic Power Prediction for Residential Load in Saudi Arabia Using Machine Learning. Energies. 2021;14(20):6759.

[45] Ajayi O, Heymann R. Data centre day-ahead energy demand prediction and energy dispatch with solar PV integration. Energy Reports. 2021;7 (11):3760–74.

[46] Wang S, Zhang Y, Hao P, Lu H. An improved method for PV output prediction using artificial neural network with overlap training range. Journal of Renewable and Sustainable Energy. 2021;13(6):063502.

[47] Nie Y, Sun Y, Chen Y, Orsini R, Brandt A. PV power output prediction from sky images using convolutional neural network: The comparison of sky-condition-specific sub-models and an end-to-end model. Journal of Renewable and Sustainable Energy. 2020;12(4):046101.

[48] Wang Y, Fu Y, Xue H. Improved prediction method of PV output power based on optimised chaotic phase space reconstruction. IET Renewable Power Generation. 2020;14(11):1831–40.

[49] Erduman A. A smart short-term solar power output prediction by artificial neural network. Electrical Engineering. 2020;102(3):1441–1449.

[50] Wang S, Zhang Y, Zhang C, Yang M. Improved artificial neural network method for predicting photovoltaic output performance. Global Energy Interconnection. 2020;3(6):553–561.

[51] Liu W, Xu Y. Randomised learning-based hybrid ensemble model for probabilistic forecasting of PV power generation. IET Generation, Transmission & Distribution. 2020;14(24):5909–5917.

[52] Chai M, Xia F, Hao S, Peng D, Cui C, Liu W. PV power prediction based on LSTM with adaptive hyperparameter adjustment. Ieee Access. 2019;7 (8):115473–86.

[53] Gamarro H, Gonzalez JE, Ortiz LE. On the assessment of a numerical weather prediction model for solar photovoltaic power forecasts in cities. Journal of Energy Resources Technology. 2019;141(6): 061203

[54] Douiri MR. A predictive model for solar photovoltaic power based on computational intelligence technique. Arabian Journal for Science and Engineering. 2019;44(8):6923–40.

[55] Liu L, Zhan M, Bai Y. A recursive ensemble model for forecasting the power output of photovoltaic systems. Solar Energy. 2019;189 (10):291–8.

[56] Gao M, Li J, Hong F, Long D. Day-ahead power forecasting in a large-scale photovoltaic plant based on weather classification using LSTM. Energy. 2019;187 (11):115838.

[57] Al-Dahidi S, Ayadi O, Adeeb J, Louzazni M. Assessment of artificial neural networks learning algorithms and training datasets for solar photovoltaic power production prediction. Frontiers in energy research. 2019;7 (11):130.

[58] Shang C, Wei P. Enhanced support vector regression based forecast engine to predict solar power output. Renewable energy. 2018;127 (11):269–83.

[59] Perveen G, Rizwan M, Goel N. Intelligent model for solar energy forecasting and its implementation for solar photovoltaic applications. Journal of Renewable and Sustainable Energy. 2018;10(6):063702.

[60] Lin P, Peng Z, Lai Y, Cheng S, Chen Z, Wu L. Short-term power prediction for photovoltaic power plants using a hybrid improved Kmeans-GRA-Elman model based on multivariate meteorological factors and historical power datasets. Energy Conversion and Management. 2018;177 (12):704–17. doi: 10.1016/j.enconman.2018.10.015

[61] Preda S, Oprea SV, Bâra A, Belciu A. PV forecasting using support vector machine learning in a big data analytics context. Symmetry. 2018;10(12):748.

[62] Alzubi JA, Kumar A, Alzubi O, Manikandan R. Efficient Approaches for Prediction of Brain Tumor using Machine Learning Techniques. Indian Journal of Public Health Research & Development. 2019;10(2): 12–21.

[63] Braik M, Al-Zoubi H, Ryalat M, Sheta A, Alzubi O. Memory based hybrid crow search algorithm for solving numerical and constrained global optimization problems. Artificial Intelligence Review. 2022: 3: 1–73.

[64] Alzubi JA, Jain R, Alzubi O, Thareja A, Upadhyay Y. Distracted driver detection using compressed energy efficient convolutional neural network. Journal of Intelligent & Fuzzy Systems. 2022;42(2):1253–65.

[65] Chinipardaz M, Amraee S. Study on IoT networks with the combined use of wireless power transmission and solar energy harvesting. Sādhanā. 2022;47(2):1–6.

[66] Hao D, Qi L, Tairab AM, Ahmed A, Azam A, Luo D, et al. Solar energy harvesting technologies for PV self-powered applications: A comprehensive review. Renewable Energy. 2022; 188 (4): 678–697.

[67] Gao D, Wu L, Hao Y, Pei G. Ultrahigh-efficiency solar energy harvesting via a non-concentrating evacuated aerogel flat-plate solar collector. Renewable Energy. 2022; 196 (8): 1455–1468.

[68] Goldvin Sugirtha Dhas B, Deepa SN. Fuzzy logic based dynamic sliding mode control of boost inverter in photovoltaic application. Journal of Renewable and Sustainable Energy. 2015;7(4):043133.

[69] Krishna SL, Jeya I, Deepa SN. Fuzzy-twin proximal SVM kernel-based deep learning neural network model for hyperspectral image classification. Neural Computing and Applications. 2022:1–34.

[70] Aruldoss Albert Victoire T. Week ahead electricity price forecasting using artificial bee colony optimized extreme learning machine with wavelet decomposition. Tehnicki vjesnik. 2021;28(2):556–67.

[71] Rani R HJ, Victoire T AA. Training radial basis function networks for wind speed prediction using PSO enhanced differential search optimizer. PloS one. 2018;13(5):e0196871. doi: 10.1371/journal.pone.0196871 29768463 PMC5955516

[72] Hannah Jessie Rani R, Aruldoss Albert Victoire T. A hybrid Elman recurrent neural network, group search optimization, and refined VMD-based framework for multi-step ahead electricity price forecasting. Soft Computing. 2019;23(18):8413–34.

[73] Chinnadurrai CL, Victoire T. Enhanced multi-objective crisscross optimization for dynamic economic emission dispatch considering demand response and wind power uncertainty. Soft Computing. 2020;24(12):9021–38.

[74] Dragomiretskiy K, Zosso D. Variational mode decomposition. IEEE transactions on signal processing. 2013;62(3):531–44.

[75] Mirjalili S. The ant lion optimizer. Advances in engineering software. 2015;83 (5):80–98.

[76] Abualigah L, Shehab M, Alshinwan M, Mirjalili S, Elaziz MA. Ant lion optimizer: a comprehensive survey of its variants and applications. Archives of Computational Methods in Engineering. 2021;28(3):1397–416.

[77] Tomar D, Agarwal S. Twin support vector machine: a review from 2007 to 2014. Egyptian Informatics Journal. 2015;16(1):55–69.

[78] Ding S, Zhang N, Zhang X, Wu F. Twin support vector machine: theory, algorithm and applications. Neural Computing and Applications. 2017;28(11):3119–30.

[79] www.kaggle.com/datasets/ef9660b4985471a8797501c8970009f36c5b3515213e2676cf40f540f0100e54?resource=download&select=Plant_2_Weather_Sensor_Data.csv –Benchmark solar PV generation datasets.

